# Effect of Reoxygenation on Radioresistance of Chronically Hypoxic A549 Non-Small Cell Lung Cancer (NSCLC) Cells Following X-Ray and Carbon Ion Exposure

**DOI:** 10.3390/ijms26189153

**Published:** 2025-09-19

**Authors:** Hasan Nisar, Bikash Konda, Marie Denise Hoffmann, Frederik M. Labonté, Maryam Arif, Sebastian Diegeler, Claudia Schmitz, Christa Baumstark-Khan, François Chevalier, Christine E. Hellweg

**Affiliations:** 1 Department of Radiation Biology, Institute of Aerospace Medicine, German Aerospace Center (DLR), 51147 Cologne, Germany; hasanisar@pieas.edu.pk (H.N.); bikash.konda@dlr.de (B.K.);; 2 Department of Medical Sciences, Pakistan Institute of Engineering and Applied Sciences (PIEAS), Islamabad 44000, Pakistan; maryamarif@pieas.edu.pk; 3 German Center for Neurodegenerative Diseases (DZNE), 53127 Bonn, Germany; 4 Department of Biology, Faculty of Mathematics and Natural Sciences, University of Cologne, 50923 Cologne, Germany; 5 Department of Pathology, University of Cambridge, Cambridge CB2 1QP, UK; 6 Université de Caen Normandie, ENSICAEN, CNRS, CEA, Normandie Université, CIMAP UMR6252, 14000 Caen, France; francois.chevalier@ganil.fr

**Keywords:** ionizing radiation, hypoxia, reoxygenation, lung cancer, survival, cell cycle, DNA double-strand breaks, DNA repair, interleukin expression, non-small cell lung cancer cells, radioresistance, epithelial–mesenchymal transition

## Abstract

Hypoxia-induced radioresistance in non-small cell lung cancer (NSCLC) hinders radiotherapy efficacy. Fractionated schedules exploit reoxygenation between fractions to reverse this resistance, but the effects of *post-irradiation* reoxygenation remain unclear and may depend on radiation quality. We investigated survival, cell cycle progression, cytokine secretion, and gene expression in hypoxic (1 % O_2_) and reoxygenated A549 cells irradiated with X-rays or carbon ions. Colony-forming assays revealed an Oxygen Enhancement Ratio (OER) > 1 for both hypoxic and reoxygenated cells after X-rays, indicating persistent radioresistance; carbon ion OER ≈ 1 reflected oxygen-independent cytotoxicity. Hypoxia weakened radiation-induced G_2_ arrest, and this was unaffected by reoxygenation. IL-6 secretion increased after X-rays and IL-8 after carbon ions exposure; both were enhanced under hypoxia and reoxygenation. RNA sequencing revealed that hypoxia induced a pro-survival, epithelial-to-mesenchymal transition (EMT)-promoting, and immune-evasive transcriptional program, which was largely reversed by reoxygenation but without increased clonogenic killing. These findings indicate that short-term reoxygenation after irradiation can normalize hypoxia-driven transcriptional changes yet does not restore radiosensitivity, supporting the advantage of high-linear energy transfer (LET) carbon ions for targeting resistant hypoxic NSCLC cells.

## 1. Introduction

Radiotherapy is used in treating over half of all cancer patients, and the response of solid tumors to radiation therapy is a key determinant of treatment efficacy in cancer management [1]. One of the most significant factors influencing radiosensitivity is the oxygenation status of tumor cells, as molecular oxygen in their nucleoplasm enhances the production of DNA-damaging reactive oxygen species (ROS) during irradiation, making it a potent radiosensitizer [2]. Tumor hypoxia, characterized by regions of low oxygen availability, poses a well-documented barrier to effective radiation therapy. As a result, hypoxic tumor cells often exhibit increased resistance to radiation-induced cell death compared to well-oxygenated cells, posing a major challenge in achieving optimal therapeutic outcomes [3].

Approximately two-thirds of X-ray-induced DNA damage occurs indirectly through ROS production. In contrast, high linear energy transfer (LET) radiation, such as carbon ions, deposits energy more densely along the tracks of radiation, leading to more direct DNA damage [4]. As a result, high-LET radiation is less dependent on ROS generation for its cytotoxic effects and may offer a therapeutic advantage in hypoxic tumors by circumventing the dependency on oxygen-mediated radiosensitization.

The Oxygen Enhancement Ratio (OER) quantifies the degree to which oxygen amplifies radiation-induced damage. It is defined as the ratio of the radiation dose needed to achieve a given biological effect under hypoxia versus normoxia [5]. For low-LET radiation, such as X-rays, the OER typically ranges from 2.5 to 3, indicating that hypoxic tumor cells may require up to three times the radiation dose to achieve the same level of cell killing as oxygenated cells. However, for high-LET radiation, the OER is lower due to the greater contribution of direct DNA damage mechanisms [6].

Beyond modulating radiosensitivity directly, hypoxia also induces a range of cellular adaptations that promote tumor cell survival, proliferation, invasion, and even metastasis, which can influence the outcome of radiotherapy. Under low-oxygen conditions, tumor cells activate hypoxia-inducible factors (HIFs), which regulate the expression of genes involved in angiogenesis, immune evasion, and metabolic reprogramming [7]. Immune evasion may protect hypoxic cells from detection and destruction by the innate and adaptive arms of the immune system [8]. Additionally, hypoxia promotes epithelial-to-mesenchymal transition (EMT), a process associated with increased invasiveness and resistance to apoptosis [9].

In tumor biology, reoxygenation refers to the process by which previously hypoxic tumor regions regain oxygen availability. This may occur following radiation treatment and represents a critical dynamic in modulating tumor radiosensitivity. For this reason, it serves as one of the rationales for fractionated radiotherapy, where the total radiation dose is delivered in multiple smaller fractions over time [10]. This approach allows for gradual reoxygenation of hypoxic tumor cells between fractions as radiation-induced cell death reduces overall metabolic demand and improves oxygen diffusion within the tumor microenvironment (TME). Consequently, previously radioresistant hypoxic cells may become more vulnerable to subsequent radiation doses [11].

However, reoxygenation has multifaceted effects on the TME. While restoring radiosensitivity via ROS generation, excessive ROS production may intensify oxidative stress, leading to DNA damage and genomic instability, which may further drive tumor progression and resistance to therapy [12]. Moreover, reoxygenation may influence cell cycle progression [13], alter cytokine secretion [14], and modulate key signaling pathways such as PI3K/AKT and MAPK, which are critical regulators of cell survival and proliferation [15,16,17]. While reoxygenation can enhance radiosensitivity, its long-term impact on tumor progression remains uncertain, warranting further investigation into its molecular consequences.

Reoxygenation prior to irradiation increases tumor radiosensitivity, and this is the key basis for fractionated radiotherapy. Due to the cellular adaptations to hypoxia described above, we hypothesized that reoxygenation after irradiation may also increase radiosensitivity by reversing the radioresistant phenotype induced by proliferative, EMT-related, and metabolic adaptations to hypoxia. Furthermore, since hypoxia influences radiosensitivity to X-rays to a higher extent than to carbon ions, we hypothesize that reoxygenation results in a stronger radiosensitization towards X-rays compared to carbon ions.

Previously, we reported that chronically hypoxic human NSCLC A549 cells (maintained at 1 % O_2_) exhibit greater radioresistance to X-rays than their normoxic counterparts, whereas this effect is significantly reduced with carbon ion irradiation [18]. We also suggested that hypoxia-induced radioresistance may be linked to alterations in cell cycle dynamics and cytokine secretion [18,19,20].

In this study, we investigated the cellular response to reoxygenation following irradiation in chronically hypoxic A549 cells. Irradiation was performed using both X-rays and carbon ions. Specifically, we compared cells that were reoxygenated post-irradiation with those that remained under hypoxic conditions. We evaluated clonogenic survival, cell cycle distribution, cytokine secretion, and gene expression. By directly comparing continuously hypoxic and reoxygenated A549 cells, our study aims to elucidate the biological mechanisms underlying NSCLC cell survival and therapeutic response after reoxygenation.

## 2. Results

**Figure 1:**
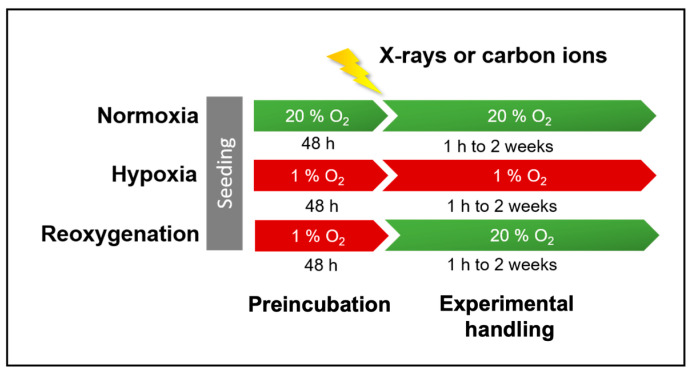
Timeline of experiments with A549 cells. Before irradiation, hypoxic cells were incubated with 1 % O_2_ and normoxic cells with 20 % O_2_ for 48 h. After irradiation, hypoxic cells were either incubated at 1 % O_2_ (continuous hypoxia) or at 20 % O_2_ (reoxygenation) during the course of all experiments.

A549 cells were pre-incubated at 20 % O_2_ (normoxia) and 1 % O_2_ (hypoxia) for 48 h prior to X-ray or carbon ion exposure (Figure 1). Following irradiation, the hypoxic cells were either maintained at 1 % O_2_ (continuous hypoxia) or 20 % O_2_ (reoxygenation).

### 2.1. Minimal Impact of Reoxygenation on Clonogenic Survival of A549 Cells After X-Ray and Carbon Ion Exposure

**Figure 2:**
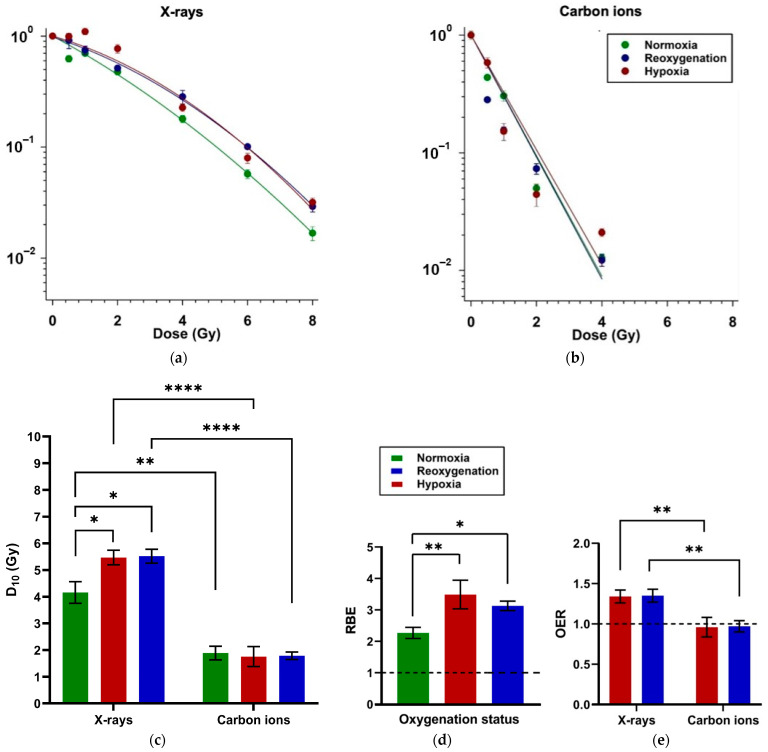
Survival of A549 cells after exposure to X-rays or carbon ions. Colony-forming ability (CFA) assay data depicting the surviving fraction as a function of dose of X-rays (**a**) and carbon ions (**b**). Cells were seeded for colony growth 24 h after irradiation. The hypoxic cells were either maintained at 1 % O_2_ after irradiation (hypoxia) or shifted to 20 % O_2_ after irradiation (reoxygenation). The radiation dose required to reduce the surviving fraction to 10 % (D_10_) is shown in (**c**). The Relative Biological Effectiveness (RBE) of carbon ions relative to X-rays under each oxygenation condition was determined based on D_10_ according to Equation (1) (see Section 4.3) (**d**). The Oxygen Enhancement Ratio (OER) at the 10 % survival level (D_10_) was calculated according to Equation (2) (see Section 4.3) (**e**) and highlights the impact of hypoxia and reoxygenation on the surviving fraction after irradiation with X-rays and carbon ions. *: *p* < 0.05; **: *p* < 0.01; ****: *p* < 0.0001; n = 6.

Continuous hypoxia (1 % O_2_ for 48 h) resulted in a higher surviving fraction of A549 cells following X-ray exposure compared to normoxia, as shown by colony formation assays (Figure 2a). This was reflected in a higher D_10_ value under hypoxia (5.47 Gy vs. 4.16 Gy under normoxia; Table A1 and Figure 2c). Reoxygenation following X-ray exposure (D_10_ = 5.52 Gy) did not significantly affect radioresistance relative to continuous hypoxia.

In contrast, after carbon ion irradiation, neither continuous hypoxia nor reoxygenation significantly altered clonogenic survival compared to normoxia. This is evident from overlapping survival curves (Figure 2b) and similar D_10_ values (Table A1 and Figure 2c).

Relative Biological Effectiveness (RBE) of carbon ions relative to X-rays was consistently > 2 across all conditions (Table A2 and Figure 2d). RBE was significantly greater under hypoxia and reoxygenation than under normoxia, though reoxygenation slightly reduced RBE compared to continuous hypoxia, without reaching statistical significance.

The Oxygen Enhancement Ratio (OER) calculated for X-rays was above 1 for both hypoxia and reoxygenation (OER = 1.34 and 1.35, respectively; Table A3 and Figure 2e), indicating hypoxia-induced radioresistance. Reoxygenation after irradiation, however, did not significantly modify this effect. OER values for carbon ions were close to 1, indicating that the presence of oxygen did not alter cell killing by carbon ions.

### 2.2. Distinct G1 and G2 Phase Radiation Responses in Reoxygenated Compared to Continuously Hypoxic A549 Cells

**Figure 3:**
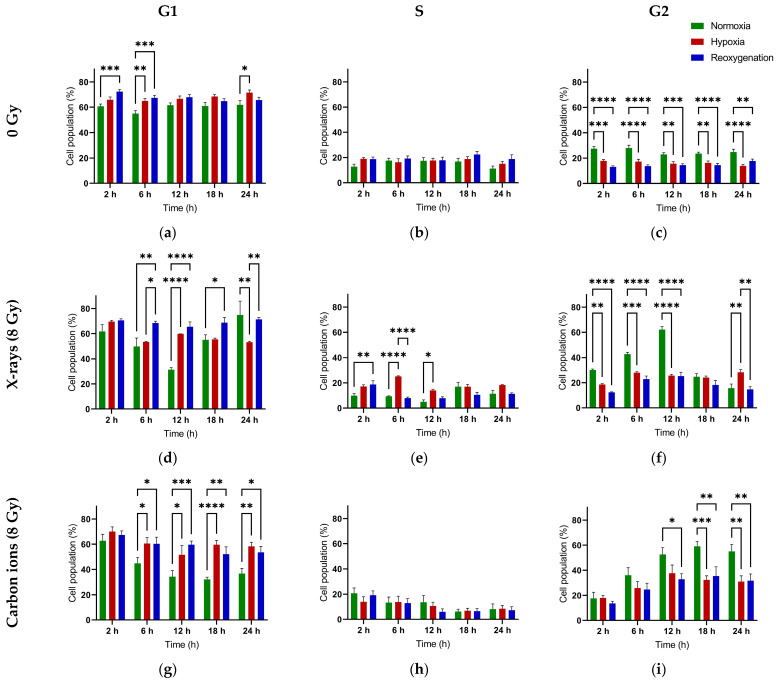
Distribution of A549 cells in the G1, S, and G2 phases of the cell cycle over time in the absence of irradiation (**a**–**c**), after exposure to X-rays—8 Gy (**d**–**f**), or carbon ions—8 Gy (**g**–**i**). Cells were pre-incubated for 48 h under normoxia (20 % O_2_) or hypoxia (1 % O_2_) and then irradiated at time 0 h. Following irradiation, the hypoxic cells were either maintained under normoxia, at 1 % O_2_ after irradiation (continuous hypoxia), or shifted to 20 % O_2_ after irradiation (reoxygenation) till the end of the experiment. *: *p* < 0.05; **: *p* < 0.01; ***: *p* < 0.001; ****: *p* < 0.0001; n = 3.

**Figure A1:**
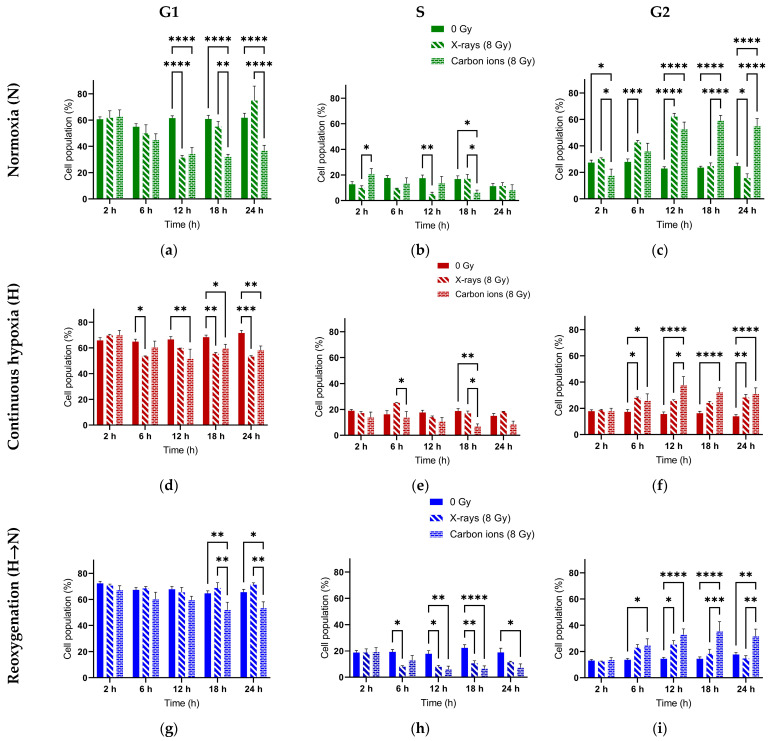
Distribution of A549 cells in the G1, S, and G2 phases of the cell cycle over time with and without exposure to ionizing radiation (X-rays and carbon ions) under normoxia (**a**–**c**), continuous hypoxia (**d**–**f**) and reoxygenation of hypoxic cells following irradiation (**g**–**i**). Cells were pre-incubated for 48 h under normoxia (20 % O_2_) or hypoxia (1 % O_2_) and then irradiated at time 0 h. Following irradiation, the hypoxic cells were either maintained at 1 % O_2_ after irradiation (continuous hypoxia) or shifted to 20 % O_2_ after irradiation (reoxygenation) till the end of the experiment. *: *p* < 0.05; **: *p* < 0.01; ***: *p* < 0.001; ****: *p* < 0.0001.

Reoxygenated A549 cells did not show a significant decline in G1 population after X-ray exposure when compared to their respective unirradiated controls, unlike normoxic and continuously hypoxic cells (Figure 3 and Figure A1). In normoxic cells, the G1 fraction returned to baseline by 18 h post-irradiation, while in continuously hypoxic cells, it remained low even at 24 h.

After carbon ion irradiation, the G1 fraction decreased in all oxygenation conditions and remained suppressed at 24 h. However, in reoxygenated cells, the decline became significant only at 18 h post-irradiation—later than in normoxic and continuously hypoxic cells (12 h).

The G1 population recovered to control levels at 24 h in normoxic and reoxygenated cells (X-rays), but not in continuously hypoxic cells. No recovery was observed in any group after carbon ion exposure. Notably, the G1 decline was significantly greater in normoxic cells compared to hypoxic or reoxygenated cells (Figure 3a,d,g).

Irradiation led to an increase in G2 population across all oxygenation states (Figure 3c,f,i). After X-rays, the G2 fraction returned to baseline in normoxic and reoxygenated cells by 18 h but remained elevated in continuously hypoxic cells even at 24 h. After carbon ion exposure, G2 remained elevated at 24 h in all groups. The G2 increase was more pronounced in normoxic cells than in hypoxic or reoxygenated cells (Figure 3f,i).

### 2.3. Minimal Modulation of IL-6 and IL-8 Secretion by Reoxygenation in A549 Cells

**Figure 4:**
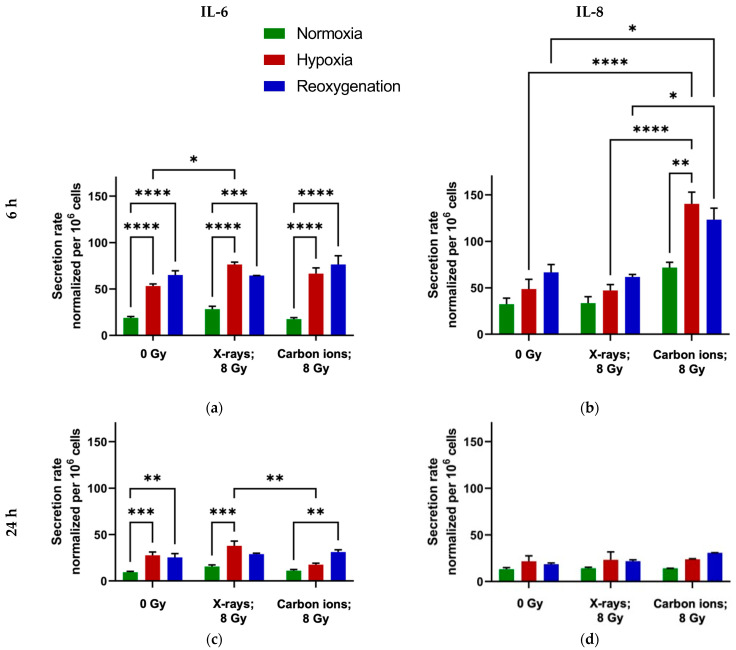
IL6 and IL-8 secretion rate (pg/h per million A549 cells), 6 h and 24 h after exposure to X-rays—8 Gy (**a**,**c**) or carbon ions—8 Gy (**b**,**d**). Cells were pre-incubated for 48 h under normoxia (20 % O_2_) or hypoxia (1 % O_2_) and then irradiated at time 0 h. Following irradiation, the hypoxic cells were either maintained at 1 % O_2_ after irradiation (hypoxia) or shifted to 20 % O_2_ after irradiation (reoxygenation) till the end of the experiment. *: *p* < 0.05; **: *p* < 0.01; ***: *p* < 0.001; ****: *p* < 0.0001; n = 3.

**Figure A2:**
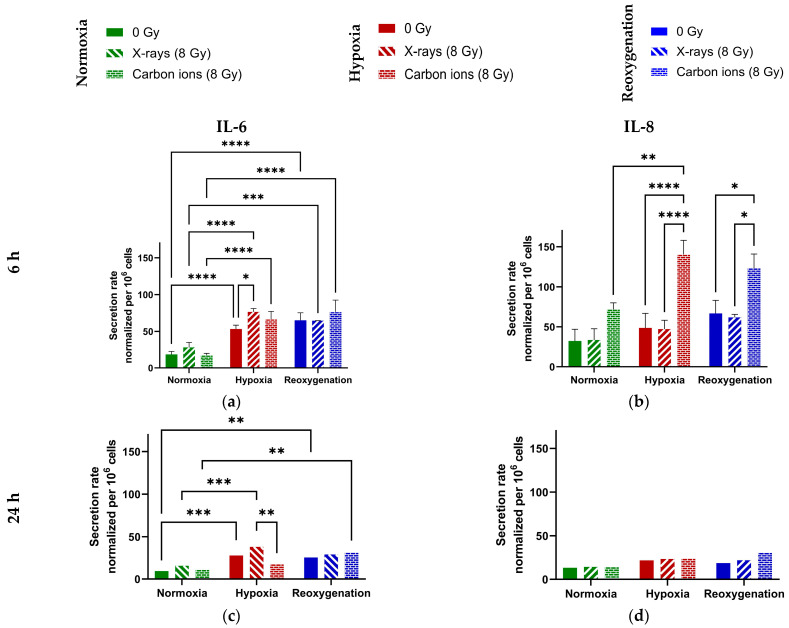
IL6 and IL-8 secretion rate (pg/h per million A549 cells), 6 h after exposure to X-rays or carbon ions (**a**,**b**), and 24 h after exposure to X-rays or carbon ions (**c**,**d**). Cells were pre-incubated for 48 h under normoxia (20 % O_2_) or hypoxia (1 % O_2_) and then irradiated at time 0 h. Following irradiation, the hypoxic cells were either maintained at 1 % O_2_ after irradiation (hypoxia) or shifted to 20 % O_2_ after irradiation (reoxygenation) till the end of the experiment. *: *p* < 0.05; **: *p* < 0.01; ***: *p* < 0.001; ****: *p* < 0.0001.

Under normoxic conditions, irradiation did not significantly alter the secretion rate of IL-6, regardless of whether X-rays or carbon ions were used (Figure A2a,c). A similar pattern was observed in reoxygenated cells, whereas continuously hypoxic cells showed a transient increase in IL-6 secretion 6 h after X-ray exposure (Figure 4a), which was not sustained at 24 h (Figure 4c).

Notably, oxygenation status itself—independent of irradiation—had a stronger effect on IL-6 secretion. Both hypoxic and reoxygenated cells exhibited significantly higher IL-6 levels compared to normoxic cells at both 6 and 24 h, with or without radiation (Figure 4a,c).

In the case of IL-8, carbon ion exposure induced a marked increase in secretion at 6 h post-irradiation in hypoxic and reoxygenated cells (Figure 4b), an effect that was not observed at 24 h (Figure 4d and Figure A2b,d) and was absent under normoxia at all time points. X-rays, by contrast, did not significantly affect IL-8 secretion under any condition.

Interestingly, while IL-6 secretion was clearly influenced by oxygenation status, IL-8 secretion was more selectively responsive to carbon ion exposure under hypoxic and reoxygenated conditions, with no significant impact from oxygenation alone.

### 2.4. Differential Gene Expression in A549 Cells Under Different Oxygenation Conditions with and Without Irradiation

To investigate the biological relevance of differential gene expression under various experimental conditions, the results were stratified into seven comparison groups:

- Continuous hypoxia without irradiation (H0) vs. normoxia without irradiation (N0)
- Reoxygenation without irradiation (R0) vs. normoxia without irradiation (N0)
- Continuous hypoxia after irradiation with 8 Gy (H8) vs. normoxia after irradiation with 8 Gy (N8)
- Reoxygenation after irradiation with 8 Gy (R8) vs. normoxia after irradiation with 8 Gy (N8)
- Normoxia after irradiation with 8 Gy (N8) vs. normoxia without irradiation (N0)
- Hypoxia after irradiation with 8 Gy (H8) vs. hypoxia without irradiation (H0)
- Reoxygenation after irradiation with 8 Gy (R8) vs. reoxygenation without irradiation (R0)

**Figure 5:**
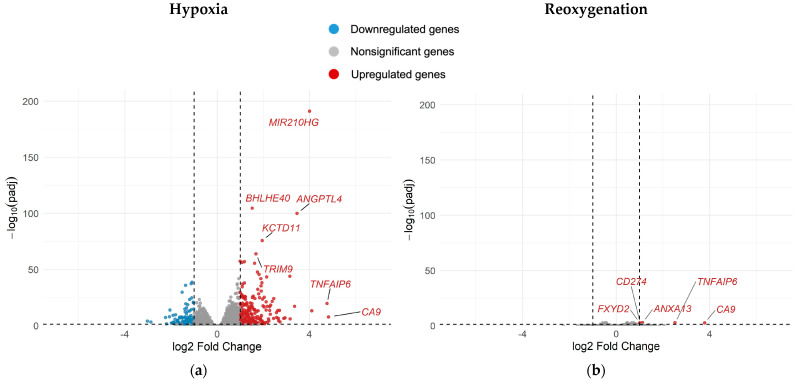
Volcano plots depicting differential gene expression in unirradiated hypoxic (H0) A549 cells relative to unirradiated normoxic (N0) cells (**a**) as well as in unirradiated reoxygenated (R0) cells relative to unirradiated normoxic (N0) cells (**b**). All cells were incubated for 52 h. Reoxygenated cells were preincubated for 48 h in hypoxia and then reoxygenated for 4 h before RNA extraction; n = 8.

The single most important finding from differential gene expression analysis was that hypoxia promoted an elaborate transcriptional program in A549 cells relative to normoxia, whereas reoxygenation largely reversed these effects. This is exemplified by volcano plots for all genes differentially regulated under hypoxia (Figure 5a) and after reoxygenation (Figure 5b).

#### 2.4.1. Reoxygenation Reversed Hypoxia-Regulated Expression of Proliferation Genes in Irradiated and Unirradiated A549 Cells

**Figure 6:**
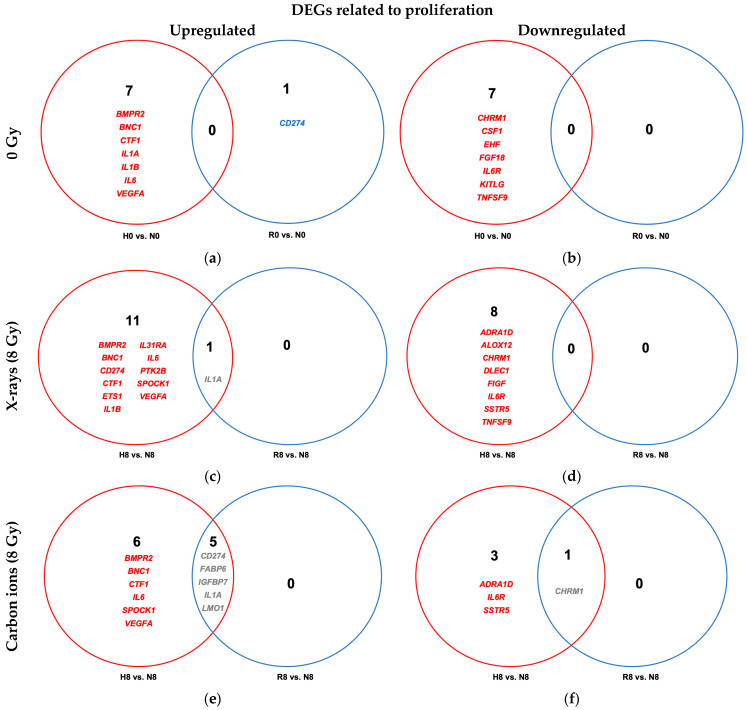
Venn diagrams of differentially expressed proliferation-related genes (GSEA M6210) in hypoxic (H) and reoxygenated (R) cells relative to normoxic (N) controls. (**a**,**b**) Differentially upregulated (**a**) and downregulated (**b**) genes in unirradiated A549 cells under hypoxia vs. normoxia (H0 vs. N0; red) and after reoxygenation vs. normoxia (R0 vs. N0; blue). (**c**,**d**) Same comparisons in cells exposed to 8 Gy X-rays under hypoxia vs. normoxia (H8 vs. N8; red) and after reoxygenation vs. normoxia (R8 vs. N8; blue). (**e**,**f**) Same comparisons in cells irradiated with 8 Gy carbon ions under hypoxia vs. normoxia (H8 vs. N8; red) and after reoxygenation vs. normoxia (R8 vs. N8; blue). All cells were incubated for 52 h, with a 4 h reoxygenation period after irradiation for the reoxygenated cells and continued hypoxia for the hypoxic cells. Sample sizes: n = 8 (unirradiated), n = 4 (irradiated).

Among the genes associated with the GSEA hallmark pathway for cell proliferation, continuous hypoxia (H0 vs. N0) led to the differential expression of 14 genes—7 upregulated and 7 downregulated compared to normoxic conditions (Figure 6a,b and Table A4). The upregulated genes included *BMPR2*, *BNC1*, *CTF1*, *IL1A*, *IL1B*, *IL6*, and *VEGFA*, many of which encode cytokines and growth factors implicated in promoting cell survival and inflammation under hypoxic stress. In contrast, *CSF1*, *TNFSF9*, *KITLG*, *EHF*, and *CHRM1* were downregulated, potentially reflecting suppression of proliferative or differentiation signals under hypoxia. This differential transcriptional response was absent in reoxygenated cells (R0 vs. N0). However, reoxygenation of hypoxic cells enhanced expression of immune evasion promoting *CD274* (PD-L1).

In irradiated A549 cells, hypoxia (H8 vs. N8, Figure 6c,d and Table A5) induced significant differential expression of multiple genes involved in promotion of proliferation and inflammation. Eight genes—*BMPR2*, *BNC1*, *CD274*, *CTF1*, *IL1A*, *IL6*, *SPOCK1*, and *VEGFA*—were upregulated, while three—*ADRA1D*, *IL6R*, and *SSTR5*—were downregulated, independent of radiation quality. Reoxygenation largely reversed these transcriptional changes, though the extent varied with radiation type: *IL1A* remained upregulated after X-ray exposure, whereas *CD274*, *FABP6*, *IGFBP7*, *IL1A*, and *LMO1* remained upregulated following carbon ion irradiation.

#### 2.4.2. Reoxygenation Reversed Hypoxia-Regulated Expression of EMT Genes in Unirradiated and Irradiated A549 Cells

**Figure 7:**
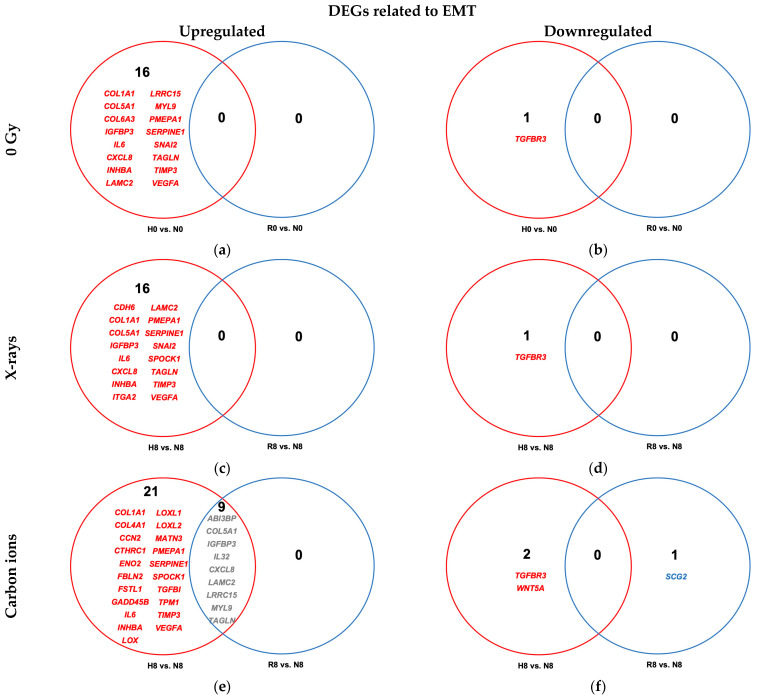
Venn diagrams of differentially expressed EMT-related genes (GSEA M5930) in hypoxic (H) and reoxygenated (R) cells relative to normoxic (N) controls. (**a**,**b**) Differentially upregulated (**a**) and downregulated (**b**) genes in unirradiated A549 cells under hypoxia vs. normoxia (H0 vs. N0; red) and after reoxygenation vs. normoxia (R0 vs. N0; blue). (**c**,**d**) Same comparisons in cells exposed to 8 Gy X-rays under hypoxia vs. normoxia (H8 vs. N8; red) and after reoxygenation vs. normoxia (R8 vs. N8; blue). (**e**,**f**) Same comparisons in cells irradiated with 8 Gy carbon ions under hypoxia vs. normoxia (H8 vs. N8; red) and after reoxygenation vs. normoxia (R8 vs. N8; blue). All cells were incubated for 52 h, with a 4 h reoxygenation period after irradiation for the reoxygenated cells and continued hypoxia for the hypoxic cells. Sample sizes: n = 8 (unirradiated), n = 4 (irradiated).

Compared to normoxic controls, continuous hypoxia (H0 vs. N0) resulted in differential upregulation of 16 EMT genes categorized in the GSEA hallmark pathway for EMT (Figure 7a,b and Table A6). These genes are known to be associated with extracellular matrix (ECM) remodeling, cytoskeletal dynamics, and modulation of cell adhesion to promote cell migration and invasiveness. Four hours of reoxygenation normalized their expression levels, making the transcriptomic EMT profile of reoxygenated cells resemble that of normoxic cells (R0 vs. N0) and reversing this effect.

In irradiated A549 cells, the comparison of hypoxic and normoxic conditions (H8 vs. N8) revealed upregulation of 16 EMT-related genes following X-ray exposure (Figure 7c,d and Table A7) and 30 genes following carbon ion exposure (Figure 7e,f and Table A7), with 13 genes commonly upregulated across both radiation qualities. Only one gene was downregulated after X-ray exposure, compared to two after carbon ion exposure. Reoxygenation (R8 vs. N8) completely reversed the hypoxia-induced EMT gene upregulation in X-ray–treated cells (Table A7); however, nine EMT-related genes remained upregulated in carbon ion–exposed cells despite reoxygenation.

#### 2.4.3. Antiproliferative Signaling Post-Irradiation Is Observed Regardless of Oxygenation Status in A549 Cells

**Figure 8:**
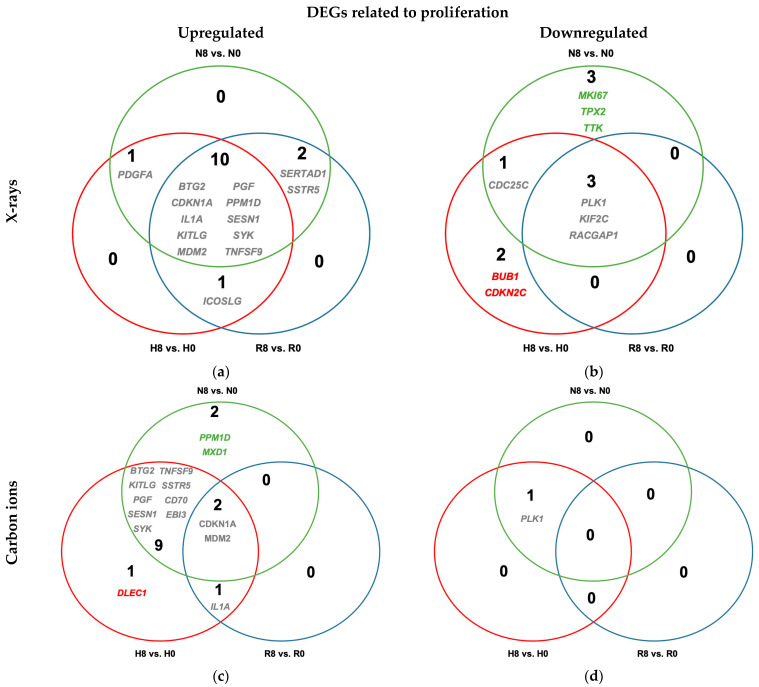
Differential upregulation (**a**,**c**) and downregulation (**b**,**d**) of genes related to proliferation in irradiated normoxic (N8), hypoxic (H8), and reoxygenated (R8) A549 cells relative to unirradiated controls (N0, H0, and R0, respectively). Cells were irradiated with either 8 Gy of X-rays (**a**,**b**) or carbon ions (**c**,**d**). All cells were incubated for 48 h prior to irradiation and for 4 h after irradiation. Differentially expressed genes in irradiated cells relative to unirradiated controls under hypoxia (H8 vs. H0) are in red, those under normoxia (N8 vs. N0) are in green, and those after reoxygenation (R8 vs. R0) are in blue; overlapping genes are listed in gray; n = 4.

Radiation exposure increased transcription of ten genes associated with proliferation control in A549 cells after X-ray exposure when compared to unirradiated controls regardless of oxygenation status (Figure 8a and Table A8). The upregulated genes included *CDKN1A* (a cyclin-dependent kinase inhibitor that enforces cell cycle arrest), *BTG2* and *SESN1* (inhibitors of cell proliferation and stress response mediators), *IL1A* (a pro-inflammatory cytokine with antiproliferative effects), *KITLG* and *PGF* (growth factors involved in cell survival and angiogenesis), *MDM2* (a p53-regulated negative feedback modulator), and *PPM1D* (a phosphatase involved in DNA damage recovery)—collectively reflecting activation of stress-adaptive and antiproliferative pathways. Additionally, *PLK1*, a key mitotic kinase, was downregulated in all conditions (Figure 8b and Table A8). This pattern suggested a net inhibitory effect on proliferation. The same trend was observed after carbon ion irradiation in normoxic and hypoxic cells (Figure 8c,d and Table A8); however, under reoxygenation following carbon ion exposure (R8 vs. R0), the transcriptional changes were not statistically significant.

#### 2.4.4. Minimal Effect of Irradiation on EMT Regulation Regardless of Oxygenation Status in A549 Cells

**Figure 9:**
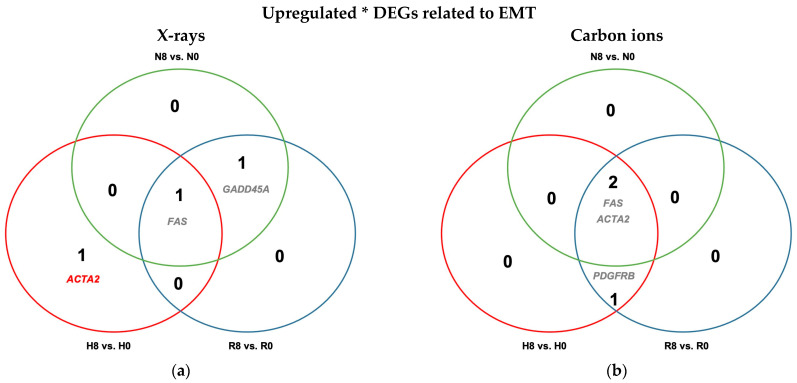
Differential upregulation of EMT-related genes in irradiated normoxic (N8), hypoxic (H8), and reoxygenated (R8) A549 cells relative to unirradiated controls (N0, H0, and R0, respectively). Cells were irradiated with either 8 Gy of X-rays (**a**) or carbon ions (**b**). All cells were incubated for 48 h prior to irradiation and for 4 h after irradiation. Differentially expressed genes in irradiated cells relative to unirradiated controls under hypoxia (H8 vs. H0) are in red, those under normoxia (N8 vs. N0) are in green, and those after reoxygenation (R8 vs. R0) are in blue; overlapping genes are listed in gray. * No downregulated DEGs related to EMT reached statistical significance; n = 4.

Evaluation of differential expression of EMT-related genes in A549 cells showed that EMT was not a major component of A549 transcriptional response to irradiation under any oxygenation condition following X-rays or carbon ion exposure (Figure 9 and Table A9).

## 3. Discussion

In this study, we examined the effect of reoxygenation on radiosensitivity and radiation response of chronically hypoxic A549 cells following irradiation with either X-rays or carbon ions. Reoxygenation after irradiation did not significantly influence clonogenic survival, cell cycle arrest, or inflammatory cytokine secretion, but it reversed key hypoxia-induced transcriptional programs.

Reoxygenation prior to irradiation is a well-established strategy to enhance tumor radiosensitivity, primarily by increasing the proportion of oxygenated cells in the radiosensitive phase of the cycle and by facilitating fixation of DNA damage in the presence of molecular oxygen. Our work deliberately diverged from this paradigm to focus on reoxygenation **after** irradiation—a scenario that can occur clinically when cytotoxic effects of radiation reduce tumor cell density and metabolic demand, thereby improving oxygen availability in the residual tumor microenvironment.

Clinically, these results may imply that while interventions aimed at increasing oxygenation remain valuable before or during irradiation, such as hyperbaric oxygen therapy, carbogen breathing, or hypoxia-targeted agents, their benefit is likely diminished if implemented only after radiation exposure. In carbon-ion therapy, high-LET tracks intrinsically mitigate hypoxia-associated radioresistance; therefore, additional reoxygenation does not add meaningful benefit irrespective of its timing.

Although reversal of hypoxia-driven programs related to EMT, inflammation, proliferation, and glucose metabolism in our study did not translate into short-term changes in radiosensitivity to a single dose of X-rays or carbon ions, it may still shape long-term tumor behavior. EMT is linked to metastasis and to cancer-stem-cell–like phenotypes associated with treatment resistance and invasiveness; likewise, hypoxia-conditioned inflammatory and proliferative signals can foster angiogenesis/lymphangiogenesis and immune evasion [21]. This is why concurrent use of antiangiogenics like bevacizumab, antiproliferative agents such as Gefitinib and immune checkpoint inhibitors such as Pembrolizumab with radiotherapy has proven to be a promising therapeutic strategy in NSCLC [22]. However, we did not assess these outcomes; future work may test whether post-irradiation reoxygenation modulates metastasis, stemness, and subsequent therapy response in vivo.

### 3.1. Reoxygenation After Irradiation Has No Impact on Survival in A549 Cells

D_10_ and OER values (Figure 2c,e) demonstrated that reoxygenation following X-ray exposure did not reverse hypoxia-induced radioresistance. Hypoxia-induced radioresistance to low-LET ionizing radiation like X-rays is well established [23] and has also been reported for NSCLC, including A549 cells [24]. Reoxygenation of hypoxic cells prior to irradiation can reverse radioresistance [25]. On the other hand, reoxygenation after irradiation has been reported to keep A549 cells radioresistant, particularly if treated concurrently with transforming growth factor β (TGF-β) [26].

On the other hand, D_10_ and OER values demonstrated no statistically significant difference in cell survival following carbon ion irradiation across all oxygenation conditions. It is fairly well established that OER of high-LET ionizing radiation like carbon ions is low, which makes them a promising therapeutic approach to treat hypoxic tumors [27,28]. The efficacy of carbon ions in killing NSCLC cells, particularly A549, has also been previously reported [28,29]. However, we were unable to find any literature evaluating the effect of reoxygenation following irradiation on the survival of irradiated hypoxic cells, which may occur clinically in both acutely and chronically hypoxic cells during radiotherapy [30,31].

The high RBE of carbon ions across all oxygenation states (Figure 2d), combined with their capacity to overcome hypoxia-induced radioresistance irrespective of reoxygenation, underscores their potential as an effective treatment option for hypoxic NSCLC cells.

### 3.2. Reoxygenation After Irradiation Has No Impact on Cell Cycle Dynamics in A549 Cells

We show that reoxygenation post-irradiation had no significant impact on G2 arrest dynamics—whether attenuation or prolongation—in hypoxic A549 cells with either radiation quality (Figure 3). However, reoxygenated cells exhibited delayed or diminished G1-phase transition within the first 24 h compared with cells maintained under hypoxia or normoxia.

The slowing of the cell cycle under chronic hypoxia is well documented, with complete arrest occurring in many cell lines under anoxic conditions [32,33]. The attenuated G2/M block observed in both continuously hypoxic and reoxygenated cells in our study may reflect reduced proliferation rates limiting the number of cells reaching the G2/M checkpoint, rather than impaired checkpoint function. The smaller attenuation observed after carbon ion exposure could be due to their ability to induce more prolonged G2 arrest (Figure 3i vs. Figure 3f), allowing more time for cells to accumulate at the checkpoint. Our findings align with previous work in hTERT-immortalized retinal pigment epithelial (RPE-1) cells, where reoxygenation did not reverse hypoxia-induced quiescence and was suggested to contribute to radioresistance in both hypoxic and reoxygenated states [34].

The delayed G1 transition we observed in reoxygenated A549 cells may result from an additional slowing of the cell cycle following irradiation. While pre-irradiation reoxygenation is generally associated with enhanced cell cycle progression [33], the immediate post-irradiation impact remains poorly characterized. Notably, hypoxia-induced cell cycle inhibition may require up to 24 h to fully reverse in HeLa cells after reoxygenation, as shown using FUCCI analysis [13].

In summary, hypoxia-induced quiescence in A549 cells persisted after 24 h of reoxygenation, potentially contributing to the relative X-ray resistance of both hypoxic and reoxygenated cells by extending the available DNA repair time [35]. In contrast, this effect appears less relevant for carbon ions, likely due to their higher RBE [35,36]. This is consistent with the established principle that quiescent cells are more resistant to low-LET radiation, such as X-rays, because they predominantly reside in the radioresistant G1 phase, whereas high-LET radiation like carbon ions is less dependent on cell cycle phase [37].

### 3.3. Reoxygenation After Irradiation Has No Impact on IL-6 and IL-8 Secretion in A549 Cells

Reoxygenation after irradiation did not significantly alter IL-6 or IL-8 secretion in hypoxic A549 cells compared with continuously hypoxic cells, irrespective of radiation quality (Figure 4).

For IL-6, hypoxia increased secretion relative to normoxia, and this difference persisted at both 6 and 24 h after irradiation and reoxygenation (Figure 4a,c). IL-6 has been linked to enhanced cell survival and proliferation via EMT activation in both normal and cancerous cells, including NSCLC [38,39,40]. Elevated IL-6 secretion in both hypoxic and reoxygenated cells may therefore contribute to the increased radioresistance observed relative to normoxic controls. The selective enhancement of IL-6 secretion under hypoxia after X-ray exposure (Figure 4a) could further increase radioresistance in hypoxic cells in our X-ray survival experiments, an effect that was absent after carbon ion irradiation.

Carbon ion irradiation significantly increased IL-8 secretion in both hypoxic and reoxygenated cells. IL-8 secretion under hypoxia has been associated with tumor cell survival through EMT activation and immune evasion via PD-L1 upregulation [41,42]. Consistent with this, our RNA sequencing data showed increased *CD274* expression (which encodes PD-L1) in irradiated hypoxic and reoxygenated cells compared with normoxic controls (Figure 6c,e). IL-8 is also induced under nutrient deprivation in A549 cells [43], suggesting that the elevated secretion observed here could promote survival under stress. However, despite higher IL-8 levels after carbon ion exposure in hypoxic or reoxygenated cells, our survival assays showed no corresponding advantage relative to normoxic counterparts.

### 3.4. Reoxygenation After Irradiation Reversed Hypoxia-Induced Transcriptional Changes

Gene expression analysis revealed that a 4 h reoxygenation period was sufficient to reverse hypoxia-induced transcriptional programs related to proliferation and EMT, with complete reversal after X-ray exposure but only partial reversal following carbon ion irradiation (Figure 7). These pathways were analyzed due to their established links to cell differentiation, cell cycle regulation, and the development of a radioresistant phenotype in hypoxic tumors.

While continuous hypoxia promoted pro-survival signaling and increased clonogenic survival after X-ray irradiation, the loss of this transcriptional profile upon reoxygenation after irradiation did not translate into reduced survival. This suggests that for radiosensitivity, the oxygen concentration and cellular status during irradiation were essential. The benefits of reoxygenation-induced transcriptional profile reversal might appear only with some time delay at a second irradiation, as exploited in clinical practice with fractionation of the total tumor dose.

For modalities such as carbon ion therapy, which inherently mitigate hypoxia-associated radioresistance through high-LET effects, this persistence is of less concern. The high RBE of carbon ions, combined with their capacity to bypass hypoxia-induced resistance irrespective of reoxygenation status, underscores their potential as an effective therapeutic option for hypoxic NSCLC tumors. Targeting such tumors with high-LET radiation may therefore circumvent the limitations of reoxygenation-based radiosensitization strategies and offer improved local control in clinically hypoxic disease.

#### 3.4.1. Reoxygenation and Proliferative Signaling

Chronic hypoxia (H0 vs. N0) in A549 cells induced a transcriptional program enriched for pro-inflammatory cytokines and growth factors (*IL1A*, *IL1B*, *IL6*, *CTF1*, *VEGFA*, *BMPR2*, *BNC1*), many linked to survival, angiogenesis, and inflammatory signaling under hypoxic stress [44,45,46] (Figure 7a,b). Simultaneously, differentiation- and immune-activating signals such as *CSF1*, *TNFSF9*, *KITLG*, *EHF*, and *CHRM1* were downregulated, potentially modulating tumor immune escape and proliferation in a context-dependent manner [47]. The overall pattern suggests that hypoxia promotes survival and immune evasion while reducing certain growth-inhibitory inputs. These changes were largely absent after reoxygenation, except for *CD274* upregulation, consistent with enhanced immune evasion potential [48,49,50].

Irradiation (both X-rays and carbon ions) consistently upregulated cell cycle inhibitors and stress-response genes such as *CDKN1A*, *BTG2*, and *SESN1* [51,52], alongside growth-promoting and survival-associated factors *KITLG*, *PGF*, *MDM2*, and *PPM1D* [53] (Figure 8). *PLK1* was uniformly downregulated, indicating mitotic suppression. This mixed profile reflects concurrent anti-proliferative checkpoint activation and pro-survival signaling, which aligns with our clonogenic assay and cell cycle analysis findings of partial but not complete proliferation suppression post-irradiation.

In irradiated cells, hypoxia maintained elevated expression of multiple pro-survival and immune-evasive genes. including *IL6*, *VEGFA*, *CD274*, *SPOCK1* [48,49,50,54,55,56,57] while suppressing genes encoding growth-inhibitory receptors *ADRA1D* and *SSTR5* [58,59,60,61] (Figure 6). Such persistence of hypoxia-driven transcription may contribute to greater radioresistance with X-rays. The effects of carbon ions, in contrast, appeared less influenced by these hypoxia-linked gene programs, consistent with their ability to overcome hypoxia-induced resistance.

Reoxygenation largely reversed hypoxia-induced transcriptional patterns, particularly after X-rays, but key immune-evasive and survival-promoting genes (e.g., *CD274*, *IL1A*) remained elevated after carbon ion exposure. The clinical relevance of these differences has to be evaluated in further studies.

Overall, chronic hypoxia primes NSCLC cells via inflammatory, angiogenic, and immune-evasive transcriptional programs; radiation triggers both growth arrest and survival-promoting DDR signals; and reoxygenation after irradiation only partially mitigates hypoxia-induced resistance—least effectively after carbon ion irradiation.

#### 3.4.2. Reoxygenation and EMT-Related Signaling

Chronic hypoxia (H0 vs. N0) in A549 cells induced a strong EMT-promoting transcriptional profile, with upregulation of 16 genes (Figure 7). Four hours of reoxygenation completely reversed this response, restoring expression to near-normoxic levels. This EMT-promoting signaling in hypoxia may contribute to radioresistance by sustaining migratory and proliferative advantages. Many hypoxia-upregulated genes, including *COL1A1*, *COL5A1*, *COL6A3*, *LAMC2*, and *SERPINE1*, are linked to enhanced migration, invasion, immune evasion, or treatment resistance in various cancers, including NSCLC [62,63,64,65,66]. Cytoskeletal regulators (*MYL9*, *TAGLN*) and transcription factors (*SNAI2*) promote EMT and cancer stemness [67,68,69], while *PMEPA1*, *IL6*, *IL8*, *IGFBP3*, *INHBA*, and *VEGFA* contribute to proliferation, angiogenesis, and immune modulation [70,71].

Following irradiation, EMT was not a dominant transcriptomic component overall (Figure 9); however, irradiated hypoxic cells (H8 vs. N8) exhibited notable EMT gene upregulation—16 genes after X-rays and 30 after carbon ions—with 13 genes common to both (Figure 7c and 7e). Reoxygenation fully reversed this after exposure to X-rays but only partially after irradiation with carbon ions, where nine EMT genes remained elevated, including *IL32*, *LOXL1*, *LOXL2*, *MATN3*, *TGFBI*, and *WNT5A*. These genes are associated with ECM remodeling, hypoxia/HIF-2 responsiveness, EMT induction, apoptosis inhibition, and radioresistance [72,73].

Overall, hypoxia primes A549 cells with a robust EMT transcriptional program that is largely reversible after reoxygenation in non-irradiated or X-ray–treated cells but is partially retained after carbon ion exposure. The persistence of EMT-related gene upregulation after carbon ions may contribute to sustained migratory, invasive, and treatment-resistant phenotypes despite restored oxygenation.

## 4. Materials and Methods

### 4.1. Cell Lines and Culture

The human lung adenocarcinoma cell line A549 (male, p53 wild type, KRAS-mutant) was procured from LGC Genomics (Berlin, Germany) [74]. Cells were maintained in 25 cm^2^ or 80 cm^2^ culture flasks (LABsolute, Th. Geyer GmbH, Renningen, Germany) using Alpha-Minimal Essential Medium (α-MEM; PAN Biotech, Aidenbach, Germany) supplemented with 10 % (*v*/*v*) dialyzed fetal bovine serum (FBS; PAN Biotech), 2 % (*v*/*v*) sterile glucose solution (0.94 mol/L), 1 % (*v*/*v*) penicillin (10,000 U/mL)/streptomycin (10 mg/mL) (PAN Biotech), 1 % (*v*/*v*) neomycin/bacitracin (Biochrom AG, Berlin, Germany), and 1 % (*v*/*v*) amphotericin B (250 µg/mL) (PAN Biotech). A seeding density of 5000 cells/cm^2^ was selected to achieve approximately 30–40 % confluence after 48 h of incubation.

Routine mycoplasma screening was performed via PCR analysis of cell culture supernatants at the Leibniz-Institut DSMZ (Braunschweig, Germany), confirming absence of contamination. Cells were cultured at 37 °C in a humidified atmosphere, either under normoxic conditions (20 % O_2_) using a CO_2_ incubator (5 % CO_2_; Heraeus HERAcell 150, Thermo Fisher Scientific, Karlsruhe, Germany) or under hypoxia (1 % O_2_) in an InvivO_2_ 400 hypoxia workstation (Baker Ruskinn, I&L Biosystems GmbH, Königswinter, Germany) flushed with 5 % CO_2_, 1 % O_2_, and 94 % N_2_.

All procedures involving hypoxic cultures—including medium replacement, fixation, and cell lysis—were carried out within the hypoxia workstation. To ensure oxygen equilibration, media and reagents were degassed by pre-warming to 25 °C in a Sonorex Digiplus ultrasonic water bath (Bandelin, Berlin, Germany) operating at 35 kHz for 40 min, followed by equilibration inside the hypoxia chamber for an additional 40 min with loosened caps prior to use.

### 4.2. Irradiation

**Figure 10:**
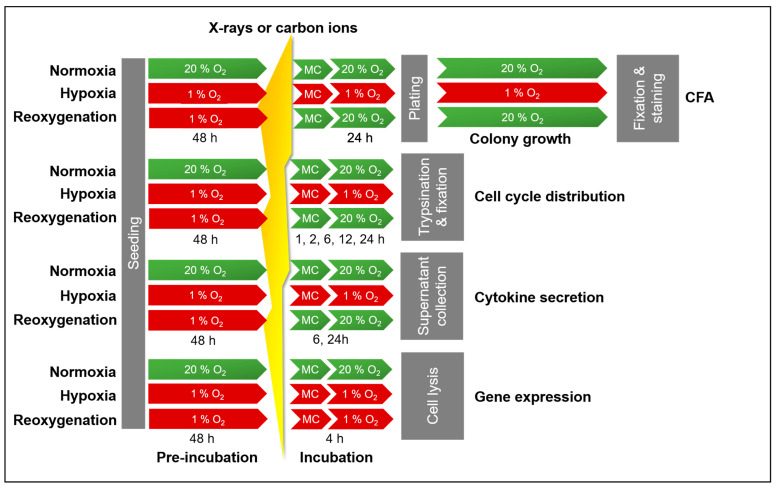
Timeline of experiments with A549 cells to determine colony-forming ability (CFA), cell cycle progression, cytokine secretion, and gene expression after exposure to X-rays or carbon ions. Before irradiation, hypoxic cells were incubated with 1 % O_2_ and normoxic cells with 20 % O_2_ for 48 h. After irradiation, subsequent handling of normoxic cells was carried out at 20 % O_2_ while that of hypoxic cells was performed at either 1 % O_2_ (continuous hypoxia) or at 20 % O_2_ (reoxygenation). MC, medium change.

Following 48 h of incubation, A549 cultures were exposed to either X-rays or carbon ions (Figure 10). Prior to irradiation, culture flask caps were securely tightened. For hypoxic samples, flasks were placed inside airtight containers before being transferred out of the hypoxia workstation via the airlock. These containers were only opened briefly during the actual irradiation period. Immediately after exposure, flasks were returned to the airtight containers and transported promptly back to the hypoxia chamber.

To confirm that this handling process did not alter oxygen levels in the culture medium, dissolved oxygen measurements were performed in preliminary trials using a Seven2Go S9 dissolved oxygen meter (Mettler Toledo, Giessen, Germany). These measurements consistently verified that oxygen concentration remained stable during transport and irradiation.

X-ray irradiation (200 kV, 15 mA; LET: 0.3–3.0 keV/µm) was performed using an RS 225 X-ray cabinet (X-strahl, Ratingen, Germany) at the Institute of Aerospace Medicine, DLR, Germany. A dose rate of 1.0 Gy/min was achieved by positioning samples 450 mm from the X-ray source exit window. To remove low-energy photons, a 0.5 mm copper filter was installed at the exit port. Cells were irradiated in either 3 cm or 6 cm culture dishes or in 25 cm^2^ or 80 cm^2^ flasks, depending on the experimental setup. Dose rate and cumulative dose were continuously monitored during exposure using a TM30013 ionization chamber connected to a UNIDOS^webline^ dosimeter (PTW, Freiburg, Germany).

Carbon ion irradiation was conducted at the cyclotron facility of the Grand Accélérateur National d’Ions Lourds (GANIL), Caen, France, at a dose rate of 1 Gy/min. Cells were positioned in the plateau region of the Bragg curve to ensure a constant LET across the cell layer. A clinically relevant LET (~75 keV/µm) in water was obtained by reducing the carbon ion beam energy from 95 MeV/n to 35 MeV/n using a 16.9 mm-thick polymethyl methacrylate (PMMA) degrader, followed by further energy loss through the polystyrene base of the culture flask. This resulted in a final beam energy of 25.7 MeV/n and a calculated LET of 73 keV/µm in water. The residual range in water was 2550 µm, confirming irradiation within the plateau region. Heavy ion fluence (P/cm^2^) was used to calculate absorbed dose (Gy). Due to the horizontal beam geometry, flasks were kept upright during exposure and filled to the neck with culture medium to avoid cell desiccation.

After irradiation, the culture medium was replaced, and cells were incubated for experiment-specific time periods. Normoxic samples were maintained at 20 % O_2_, while hypoxic samples were either kept at 1 % O_2_ (continuous hypoxia) or switched to 20 % O_2_ (reoxygenation).

### 4.3. Cell Survival Analysis Following Irradiation Under Normoxia and Hypoxia

Clonogenic survival was assessed using Puck’s colony-forming assay (CFA) to compare surviving fractions of A549 cells maintained under normoxia, continuous hypoxia, or reoxygenation (Figure 10) after receiving graded doses of X-rays (0, 0.5, 1, 2, 4, 6, and 8 Gy) or carbon ions (0, 0.5, 1, 2, and 4 Gy).

Cells were seeded in 25 cm^2^ flasks and pre-incubated for 48 h under either normoxic (20 % O_2_) or hypoxic (1 % O_2_) conditions before irradiation (Section 4.2). Following irradiation or sham exposure, the culture medium was immediately replaced. Continuous hypoxia samples were returned to the hypoxia workstation, whereas normoxic samples were placed back in the standard incubator. For reoxygenation, a subset of hypoxic cells was transferred to normoxic conditions (20 % O_2_).

Twenty-four hours post-irradiation, cells were trypsinized and replated in 6 cm Petri dishes (LABsolute, Th. Geyer GmbH, Renningen, Germany) for colony formation. The number of cells seeded was adjusted according to plating efficiency and the expected dose-dependent cell killing to yield approximately 75 colonies per dish. Normoxic and reoxygenated cultures were handled and incubated under standard conditions (20 % O_2_) for two weeks. Hypoxic cultures were replated and grown within the hypoxia workstation at 1 % O_2_ for the same duration. Colonies were fixed and stained for counting after the incubation period (Figure 10).

After two weeks, once the colonies became visible, they were fixed and stained with 5 mL of crystal violet (0.2 % *w*/*v*)–formaldehyde (3.5 %) staining solution per Petri dish for 20 min after removing culture medium from the Petri dishes. Stained colonies comprising over 50 cells were counted using a manual colony counter (Schuett count, Schuett-biotec, Göttingen, Germany). Survival fractions were determined by dividing the colony count by the number of cells that were seeded for each dose. Survival curves were generated for each oxygen condition and radiation quality by plotting the surviving fractions on a logarithmic scale as a function of dose on a linear scale. The single-hit multi-target model was used to perform regression analysis of experimental data. In addition, we derived D_10_—the radiation dose that resulted in 10 % survival—from the modeled curves.

The Relative Biological Effectiveness (RBE) of carbon ions compared to X-rays under different oxygen conditions was calculated according to Equation (1):

(1) $$RBE=\frac{D_{10\;(X-rays)}}{D_{10\;(Carbon\;ions)}}$$

The influence of hypoxia and of reoxygenation after chronic hypoxia on cell survival was evaluated by calculating the Oxygen Enhancement Ratio (OER) using Equations (2) and (3), respectively:

(2) $$Hypoxia:OER=\frac{D_{10\;(Continuous\;Hypoxia)}}{D_{10\;(Normoxia)}}$$

(3) $$Reoxygenation:OER=\frac{D_{10\;(Reoxygenation)}}{D_{10\;(Normoxia)}}$$

### 4.4. Analysis of Cell Cycle Response Following X-Ray Exposure Under Normoxia and Hypoxia

A549 cells were seeded in 6 cm Petri dishes at a density of 5000 cells/cm^2^ and incubated for 48 h under either normoxic (20 % O_2_) or hypoxic (1 % O_2_) conditions. Cells were then exposed to either X-rays or carbon ions at a dose of 8 Gy. After irradiation, normoxic cultures were returned to 20 % O_2_, while hypoxic cultures were either maintained at 1 % O_2_ (continuous hypoxia) or shifted to 20 % O_2_ (reoxygenation) (Figure 10).

At selected time points over the subsequent 24 h, cells were harvested by trypsin/EDTA detachment (1 mL) and fixed in 3.5 % formaldehyde. After 30 min, fixed cells were washed with phosphate-buffered saline (PBS) and stained with 4′,6-diamidino-2-phenylindole (DAPI; 500 ng/mL) containing Triton X-100 (3 µg/mL) in PBS. Samples were incubated for 30 min in the dark at room temperature.

DNA content was quantified using a Cytoflex S flow cytometer (Beckman Coulter, Indianapolis, IN, USA). DAPI was excited with a violet laser (405 nm), and fluorescence emission was collected in the PB450 channel. Cell cycle phase distribution for normoxic, continuously hypoxic, and reoxygenated samples was determined after applying gates on forward vs. side scatter and PB450 width vs. area plots to exclude debris and doublets. Histograms of single-cell fluorescence were analyzed using FloJo software (Version 10, BD Biosciences, San Jose, CA, USA) with the Dean–Jett–Fox mathematical model [75].

### 4.5. Quantification of Cytokine Secretion Following X-Ray Exposure Under Normoxia and Hypoxia

The concentrations of IL-6 and IL-8 in culture supernatants were determined using ELISA kits (Invitrogen, Thermo Fisher Scientific, Karlsruhe, Germany). Supernatants (3 mL) were collected at 6 h and 24 h after irradiation with either X-rays or carbon ions. Following collection, samples were placed in Eppendorf tubes and stored at −80 °C until analysis. Normoxic samples were re-incubated at 20 % O_2_, whereas hypoxic samples were either maintained at 1 % O_2_ (continuous hypoxia) or transferred to 20 % O_2_ (reoxygenation) (Figure 10).

For normalization, A549 cell counts from each corresponding culture were obtained using the LUNA automated cell counter after detachment with 3 mL trypsin/EDTA.

Ninety-six–well ELISA plates (Corning™ Costar™ 9018, Kaiserslautern, Germany) were coated with primary capture antibodies (100 µL/well, 1:250 dilution in PBS) provided in the kit and incubated overnight at 4 °C. Wells were then blocked with 200 µL/well blocking buffer (1:5 dilution in deionized water) to prevent non-specific binding. Samples (100 µL/well) and serial dilutions of the supplied standard were added to the plates, followed by overnight incubation at 4 °C.

Detection antibodies (100 µL/well, 1:250 dilution in PBS) from the kit were then applied, followed by 1 h incubation at room temperature. For IL-6 detection, Streptavidin-HRP (100 µL/well, 1:100 dilution) was used; for IL-8 detection, Avidin-HRP (100 µL/well, 1:250 dilution) was applied. Plates were incubated for 30 min at room temperature before adding the substrate 3,3′,5,5′-Tetramethylbenzidine (TMB) (100 µL/well) for 15 min. The reaction was stopped with 100 µL/well of 2 N H_2_SO_4_.

All incubations were performed on a plate shaker, with five washes between steps using PBS containing 0.05 % Tween-20.

### 4.6. Gene Expression Analysis Following X-Ray Exposure Under Normoxia and Hypoxia

Global transcriptional profiles were assessed in A549 cells cultured under normoxia, continuous hypoxia, or reoxygenation following exposure to 8 Gy of X-rays or carbon ions (Figure 10). Four hours post-irradiation, the culture medium was completely removed, and cells were lysed in RLT buffer (Qiagen, Hilden, Germany) supplemented with β-mercaptoethanol (1:100; Sigma-Aldrich, St. Louis, MO, USA). Total RNA was extracted using the RNeasy Mini Kit (Qiagen) according to the manufacturer’s instructions.

RNA concentration and integrity were determined using the RNA 6000 Nano Assay (Agilent Technologies, Böblingen, Germany) on a Bioanalyzer (Agilent Technologies). Only samples with RNA Integrity Numbers (RIN) > 9.0 and yielding at least 3 µg total RNA (n = 4 biological replicates per condition) were processed further.

Samples were shipped on dry ice to GENEWIZ (Leipzig, Germany) for mRNA sequencing. Poly(A)-selected libraries were prepared and sequenced on the Illumina NovaSeq 6000 platform (paired-end, 2 × 150 bp; ~350 million read pairs per run). Reads were aligned to the *Homo sapiens* GRCh38 reference genome, and unique gene hit counts within exon regions were obtained. Differential expression analysis was performed in R using the DESeq2 package version 1.48.2 [76]. Genes were classified as differentially expressed if they met both criteria: adjusted *p*-value < 0.05 and absolute log_2_ fold change > 1.

To investigate biological relevance, differential expression results were stratified into predefined comparison groups as shown in Table 1.

Within each comparison group, we focused on differential expression changes in curated gene panels associated with proliferation, epithelial–mesenchymal transition (EMT), and glycolysis. The EMT panel was defined using the GSEA hallmark gene set M5930 (200 genes), and proliferation using the GSEA hallmark pathway M16210 (512 genes).

### 4.7. Statistical Analysis

All experiments were performed at least three times (RNA Seq four times) at independent time points, with technical replicates varying by assay type: six for colony-forming assays, four for RNA sequencing, and three for cell cycle and cytokine analyses. Data processing, including calculation of means, standard deviations, and standard errors of the mean (SE), was carried out in Microsoft Excel 2016 (Microsoft Corporation, Redmond, WA, USA). Graph generation and statistical testing were performed using GraphPad Prism 9 (Dotmatics, Boston, MA, USA). For survival data obtained from colony-forming assays, multiple two-way unpaired *t*-tests were applied. Cell cycle and cytokine datasets were evaluated by two-way ANOVA. RNA sequencing data were analyzed using the Wald test to calculate *p*-values, with false discovery rate (FDR) adjustment via the Benjamini–Hochberg method to obtain adjusted *p*-values (padj).

## 5. Conclusions

Our findings reveal a nuanced interaction between chronic hypoxia, reoxygenation, and radiation quality in shaping the biological response of A549 NSCLC cells. High-LET carbon ions consistently overcame hypoxia-induced radioresistance, whereas X-rays retained reduced cytotoxicity under hypoxia that was not restored by immediate post-irradiation reoxygenation. Reoxygenation after irradiation did not enhance radiosensitivity, alter hypoxia-attenuated G2/M arrest, or modify inflammatory cytokine secretion, yet it substantially reversed hypoxia-driven transcriptional programs related to proliferation and epithelial–mesenchymal transition. The persistence of hypoxia-associated functional resistance despite transcriptional normalization suggests that it occurs too late restore radiosensitivity in chronically hypoxic tumors for an irradiation occurring just before reoxygenation, but the normalization might be beneficial in controlling invasive and metastatic behavior of the tumor cells and in reducing radioresistance when the next radiotherapy fraction is applied. Clinically, while oxygen-modifying strategies remain valuable when applied before or during irradiation, their benefit appears limited after radiation exposure. High-LET modalities such as carbon ion therapy may therefore provide a more robust approach for targeting hypoxic NSCLC subpopulations, independent of reoxygenation status.

## Figures and Tables

**Table 1:** Comparison groups for evaluation of differential gene expression in A549 cells following modulation of oxygenation and irradiation status (X-rays/carbon ions).

Effect Being Studied	Abbreviation	Compared Groups
Effect of oxygenation status in unirradiated cells	H0 vs. N0	Continuous hypoxia without irradiation vs. normoxia without irradiation
	R0 vs. N0	Reoxygenation after 48 h of hypoxia without irradiation vs. normoxia without irradiation
Effect of oxygenation status in irradiated cells	H8 vs. N8	Hypoxia before and after irradiation with 8 Gy vs. normoxia before and after irradiation with 8 Gy
	R8 vs. N8	Reoxygenation after 48 h of hypoxia and irradiation with 8 Gy vs. normoxia before and after irradiation with 8 Gy
Effect of irradiation under different oxygen conditions	N8 vs. N0	Normoxia before and after irradiation with 8 Gy vs. normoxia without irradiation
	H8 vs. H0	Hypoxia before and after irradiation with 8 Gy vs. hypoxia without irradiation
	R8 vs. R0	Reoxygenation after 48 h of hypoxia and irradiation with 8 Gy vs. reoxygenation without irradiation

**Figure A3:**
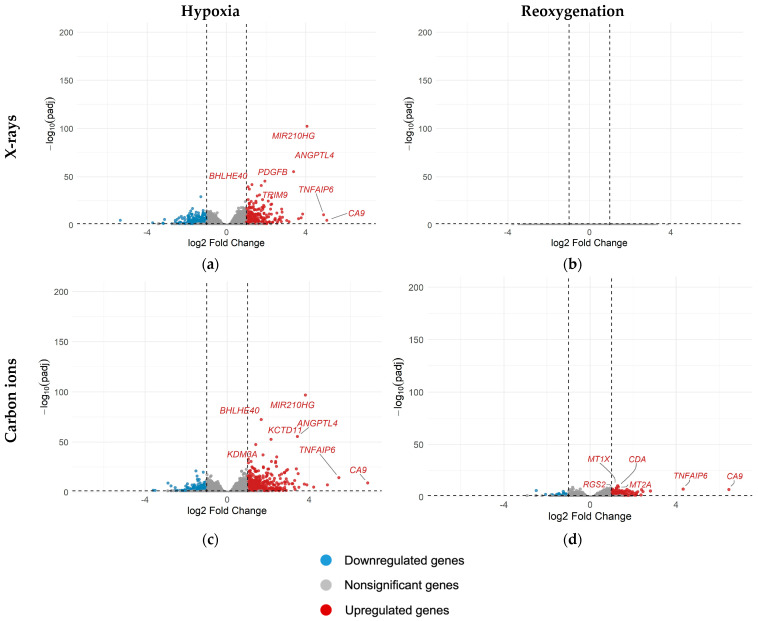
Volcano plots depicting differentially upregulated and downregulated genes in irradiated A549 cells under hypoxia relative to normoxia (H8 vs. N8) with either X-rays (**a**) or carbon ions (**c**); or in those A549 cells reoxygenated after irradiation relative to those irradiated under normoxia (R8 vs. N8), either with X-rays (**b**) or carbon ions (**d**).

## Data Availability

Research data are stored in an institutional repository and will be shared upon request to the corresponding author.

## Abbreviations

The following abbreviations are used in this manuscript (gene name abbreviations are explained in Table A10):

α-MEM	Alpha-Minimally Essential Medium
ANOVA	Analysis of Variance
ARE	Antioxidative Response Element
CFA	Colony-Forming Ability
DAPI	4′,6-Diamidino-2-Phenylindole
DSMZ	German Collection of Microorganisms and Cell Cultures GmbH
ECM	Extracellular Matrix
EDTA	Ethylene Diamine Tetra Acetic Acid
EGFR	Epidermal Growth Factor Receptor
ELISA	Enzyme-Linked Immunosorbent Assay
EMT	Epithelial–Mesenchymal Transition
FBS	Fetal Bovine Serum
FDR	false discovery rate
FUCCI	Fluorescent Ubiquitination-based Cell Cycle Indicator
HIFs	hypoxia-inducible factors
hTERT	human telomerase reverse transcriptase
LET	Linear Energy Transfer
NF-κB	Nuclear Factor κB
NSCLC	Non-Small Cell Lung Cancer
OER	Oxygen Enhancement Ratio
PBS	Phosphate-buffered saline
PMMA	Polymethyl methacrylate
RBE	Relative Biological Effectiveness
RNA	Ribonucleic acid
ROS	Reactive Oxygen Species
RPE-1	Human Retinal Pigment Epithelial-1
RIN	RNA Integrity Numbers
TGF-β	transforming growth factor β
TMB	3,3′,5,5′-Tetramethylbenzidine
TME	Tumor Microenvironment

## Appendix A

### Appendix A.1. Cell Survival—Characteristics

**Table A1:** Dose required to reduce the surviving fraction to 10 % (D_10_) of the pre-irradiation cell number after exposure of A549 cells to X-rays or carbon ions under various oxygen conditions.

Radiation Quality	Plating Time	D_10_ (Gy) (±SE)			*p*-Values		
		Normoxia (N)	Hypoxia (H)	Reoxygenation (R: H→N)	Normoxia vs. Hypoxia	Normoxia vs. Reoxygenation	Reoxygenation vs. Hypoxia
X-rays	LP	4.16 ± 0.40	5.47 ± 0.27	5.52 ± 0.26	0.039	0.029	ns
Carbon ions	LP	1.89 ± 0.25	1.76 ± 0.38	1.79 ± 0.14	ns	ns	ns

D_10_ was derived from the survival curves generated by colony-forming ability (CFA) assays (Figure 2); LP: late plating; ns: not significant.

**Table A2:** Relative Biological Effectiveness (RBE) based on D_10_.

Radiation Quality	Type of Assay	RBE (±SE)			*p*-Values		
		Normoxia (N)	Hypoxia (H)	Reoxygenation (R: H→N)	N vs. H	N vs. R	R vs. H
Carbon ions compared to X-rays	LP	2.27 ± 0.18	3.49 ± 0.46	3.13 ± 0.15	0.002	0.039	ns

LP: late plating; ns: not significant.

**Table A3:** Oxygen Enhancement Ratio (OER) based on D_10_.

Radiation Quality	Type of Assay	OER (±SE)		*p*-Values
		Hypoxia (H)	Reoxygenation (R: H→N)	H vs. R
X-rays	Late plating	1.34 ^a^ ± 0.08	1.35 ^b^ ± 0.08	ns
Carbon ions	Late plating	0.96 ± 0.12	0.97 ± 0.07	ns

ns: not significant; ^a^ for X-rays vs. carbon ions exposure under hypoxia, *p*-value = 0.001; ^b^ for X-rays vs. carbon ions exposure after reoxygenation, *p*-value = 0.001.

### Appendix A.4. Differential Gene Expression

**Table A4:** Effect of oxygen conditions on expression of cellular proliferation genes in continuously hypoxic (H) A549 cells relative to normoxic (N) controls and in reoxygenated (R) A549 cells relative to normoxic (N) controls.

NCBI Gene Symbol	Log_2_ Fold Change		NCBI Gene Name	Log_2_ Fold Change	
	H0 ^1^ vs. N0	R0 vs. N0		H0 ^1^ vs. N0	R0 vs. N0
*BMPR2*	**1.12**	0.16	IL1A	**2.63**	0.93
*BNC1*	**1.15**	0.31	IL1B	**1.80**	0.43
*CD274*	0.91	**1.03**	IL6	**1.23**	0.67
*CHRM1*	**−3.02**	−0.01	IL6R	**−1.36**	−0.36
*CSF1*	**−1.08**	−0.39	KITLG	**−1.10**	−0.19
*CTF1*	**1.23**	0.42	TNFSF9	**−1.09**	−0.10
*EHF*	**−1.40**	−0.01	VEGFA	**1.00**	0.19
*FGF18*	**−1.05**	−0.86			

^1^ H0 signifies unirradiated, continuous hypoxic (1 % O_2_) A549 cells, N0 represents unirradiated normoxic cells, and R0 denotes unirradiated A549 cells that were reoxygenated after 48 h of hypoxia (1 % O_2_). All cells were preincubated for 48 h in specified oxygen conditions, followed by a medium change. RNA isolation from cells was carried out 4 h after the medium change; ns: not significant; Statistically significant results are in bold; in red: upregulation, in blue: downregulation. The GSEA hallmark pathway for cell proliferation (M16210) was utilized to acquire proliferation-related genes.

**Table A5:** Combined effect of oxygen concentration and irradiation on the regulation of proliferation-related genes in irradiated, continuously hypoxic A549 cells (H8) relative to irradiated normoxic A549 cells (N8), and in hypoxic A549 cells that were reoxygenated after irradiation (R8) relative to irradiated, normoxic cells (N8).

NCBI Gene Symbol	Log_2_ Fold Change			
	H8 ^1^ vs. N8		R8 vs. N8	
	X-Rays	Carbon Ions	X-Rays	Carbon Ions
*ADRA1D*	**−1.03**	**−1.19**	−0.32	−0.78
*ALOX12*	**−1.24**	−0.30	−0.21	−0.06
*BMPR2*	**1.25**	**1.15**	0.04	0.29
*BNC1*	**1.87**	**1.04**	0.49	0.96
*CD274*	**1.11**	**1.05**	0.59	**1.56**
*CHRM1*	**−5.36**	**−3.60**	−0.99	**−2.92**
*CTF1*	**1.81**	**1.09**	1.49	−0.02
*DLEC1*	**−1.19**	0.10	−0.02	−0.25
*ETS1*	**1.05**	0.76	0.30	0.56
*FABP6*	0.13	**1.17**	−0.52	**1.20**
*FIGF*	**−1.38**	−0.79	−0.37	−0.23
*IGFBP7*	0.69	**1.29**	0.09	**1.09**
*IL1A*	**2.81**	**3.46**	**1.13**	**2.40**
*IL1B*	**2.58**	0.05	0.96	N/A
*IL31RA*	**1.08**	0.58	0.22	0.32
*IL6*	**1.18**	**1.28**	0.50	0.96
*IL6R*	**−1.18**	**−1.05**	−0.24	−0.51
*LMO1*	0.40	**1.56**	0.00	**1.17**
*PTK2B*	**1.01**	0.31	0.25	0.13
*SPOCK1*	**1.05**	**1.06**	0.30	0.81
*SSTR5*	**−1.64**	**−1.36**	0.04	−1.12
*TNFSF9*	**−1.05**	−0.95	−0.16	−0.58
*VEGFA*	**1.09**	**1.04**	0.10	−0.09

^1^ H8, N8, and R8 signify continuously irradiated hypoxic (1 % O_2_), normoxic, and reoxygenated A549 cells, respectively. All cells were preincubated for 48 h in specified oxygen conditions, followed by irradiation and a medium change. RNA isolation from cells was carried out 4 h after irradiation. Statistically significant results are in bold; in red: upregulation, in blue: downregulation. The GSEA hallmark pathway for cell proliferation (M16210) was utilized to acquire proliferation-related reference genes.

**Table A6:** Effect of oxygen concentration on expression of EMT-related genes in continuously hypoxic (H) A549 cells relative to normoxic (N) controls and in reoxygenated (R) A549 cells relative to normoxic (N) controls.

NCBI Gene Symbol	Log_2_ Fold Change		NCBI Gene Name	Log_2_ Fold Change	
	H0 ^1^ vs. N0	R0 vs. N0		H0 ^1^ vs. N0	R0 vs. N0
*COL1A1*	**1.80**	0.35	MYL9	**1.18**	0.67
*COL5A1*	**1.43**	0.54	PMEPA1	**1.18**	0.17
*COL6A3*	**1.13**	0.35	SERPINE1	**1.64**	0.43
*IGFBP3*	**1.73**	0.75	SNAI2	**1.04**	0.60
*IL6*	**1.23**	0.67	TAGLN	**1.21**	0.56
*CXCL8*	**1.31**	0.84	TGFBR3	**−1.52**	−0.32
*INHBA*	**1.41**	0.16	TIMP3	**1.35**	0.70
*LAMC2*	**1.64**	0.44	VEGFA	**1.00**	0.19
*LRRC15*	**1.58**	0.32			

^1^ H0 signifies unirradiated, continuous hypoxic (1 % O_2_) A549 cells, N0 represents unirradiated normoxic cells, and R0 denotes unirradiated A549 cells that were reoxygenated after 48 h of hypoxia (1 % O_2_). All cells were preincubated for 48 h in specified oxygen conditions, followed by a medium change. RNA isolation from cells was carried out 4 h after the medium change. Statistically significant results are in bold; in red: upregulation, in blue: downregulation. The GSEA hallmark pathway for epithelial–mesenchymal transition (M5930) was utilized to acquire EMT reference genes.

**Table A7:** Combined effect of oxygen concentration and irradiation on the expression of EMT-related genes in irradiated, continuously hypoxic A549 cells (H8) relative to irradiated normoxic A549 cells (N8), and in hypoxic A549 cells that were reoxygenated after irradiation (R8) relative to irradiated, normoxic A549 cells (N8).

NCBI Gene Symbol	Log_2_ Fold Change			
	H8 ^1^ vs. N8		R8 vs. N8	
	X-Rays	Carbon Ions	X-Rays	Carbon Ions
*ABI3BP*	0.41	**1.21**	0.11	**1.09**
*CDH6*	**1.33**	0.88	0.42	0.10
*COL1A1*	**1.75**	**2.36**	0.32	0.89
*COL4A1*	0.89	**1.02**	0.03	0.54
*COL5A1*	**1.51**	**1.95**	0.24	**1.35**
*CCN2*	1.30	**1.18**	-0.16	0.19
*CTHRC1*	0.11	**1.37**	-0.29	0.80
*ENO2*	0.56	**1.08**	0.01	0.51
*FBLN2*	0.40	**1.07**	-0.10	0.72
*FSTL1*	0.91	**1.01**	0.09	0.74
*GADD45B*	0.93	**1.19**	0.21	0.66
*IGFBP3*	**1.36**	**2.82**	0.32	**1.95**
*IL32*	0.72	**1.40**	0.12	**1.45**
*IL6*	**1.18**	**1.28**	0.50	0.96
*CXCL8*	**1.49**	**1.41**	0.65	**1.27**
*INHBA*	**1.55**	**1.55**	0.24	0.74
*ITGA2*	**1.61**	0.69	0.25	0.50
*LAMC2*	**2.16**	**1.42**	0.37	**1.03**
*LOX*	0.37	**1.07**	0.08	0.37
*LOXL1*	0.53	**1.25**	0.14	1.08
*LOXL2*	0.70	**1.18**	0.18	0.87
*LRRC15*	0.97	**2.81**	-0.08	**1.55**
*MATN3*	0.54	**1.09**	0.25	0.89
*MYL9*	0.73	**1.87**	0.12	**1.59**
*PMEPA1*	**1.25**	**1.25**	0.09	0.40
*SCG2*	−0.53	−0.99	−0.03	**−1.07**
*SERPINE1*	**1.84**	**1.91**	0.29	0.98
*SNAI2*	**1.03**	0.95	0.44	0.72
*SPOCK1*	**1.05**	**1.06**	0.30	0.81
*TAGLN*	**1.15**	**2.02**	0.04	**1.71**
*TGFBI*	0.88	**1.36**	0.02	0.81
*TGFBR3*	**−1.19**	**−1.36**	−0.17	−0.62
*TPM1*	0.44	**1.22**	−0.02	0.98
*TIMP3*	**1.47**	**2.26**	0.19	1.30
*VEGFA*	**1.09**	**1.04**	0.10	−0.09
*WNT5A*	−0.42	**−1.10**	0.06	−0.65

^1^ H8, N8, and R8 signify continuously irradiated hypoxic (1 % O_2_), normoxic, and reoxygenated A549 cells, respectively. All cells were preincubated for 48 h in specified oxygen conditions, followed by irradiation and a medium change. RNA isolation from cells was carried out 4 h after irradiation. Statistically significant results are in bold; in red: upregulation, in blue: downregulation. The GSEA hallmark pathway for epithelial–mesenchymal transition (M5930) was utilized to acquire EMT reference genes.

**Table A8:** Effect of ionizing radiation on expression of proliferation-related genes by normoxic A549 cells (N), continuously hypoxic (1 % O_2_) A549 cells (H), and hypoxic A549 cells reoxygenated after irradiation (R). Differential expression refers to irradiated (N8, H8, R8) versus unirradiated (N0, H0, R0) cells.

NCBI Gene Symbol	Log_2_ Fold Change					
	N8 ^1^ vs. N0		H8 vs. H0		R8 vs. R0	
	X-Rays	Carbon Ions	X-Rays	Carbon Ions	X-Rays	Carbon Ions
*BTG2*	**2.70**	**1.97**	**2.71**	**1.83**	**2.74**	1.88
*CDKN1A*	**2.63**	**2.61**	**2.62**	**2.38**	**2.56**	**2.50**
*IL1A*	**1.37**	0.43	**1.54**	**1.26**	**1.57**	**1.90**
*KITLG*	**1.01**	**1.36**	**1.38**	**1.46**	**1.06**	1.27
*MDM2*	**2.47**	**2.14**	**2.65**	**2.16**	**2.37**	**2.12**
*PGF*	**2.46**	**2.59**	**2.65**	**3.05**	**2.50**	2.94
*PLK1*	**−1.73**	**−1.59**	**−2.01**	**−1.61**	**−1.57**	−1.65
*PPM1D*	**2.03**	**1.47**	**1.90**	1.23	**1.91**	1.25
*SESN1*	**2.31**	**2.06**	**2.32**	**1.95**	**2.33**	2.07
*SYK*	**1.40**	**1.58**	**1.06**	**1.26**	**1.37**	0.87
*TNFSF9*	**1.53**	**1.39**	**1.57**	**1.52**	**1.47**	0.91
*SSTR5*	**1.38**	**1.62**	0.64	**1.16**	**1.64**	0.72
*BUB1*	−0.95	−0.81	**−1.04**	−0.99	−0.79	−0.97
*CD70*	0.70	**1.06**	0.91	**1.10**	0.77	1.13
*CDC25C*	**−1.03**	−0.62	**−1.03**	−0.69	−0.77	−0.75
*CDKN2C*	−0.76	−0.98	**−1.08**	−0.66	−0.83	−0.88
*DLEC1*	0.11	0.47	−0.22	**1.44**	0.19	0.33
*EBI3*	0.60	**1.04**	0.81	**1.45**	0.16	1.02
*ICOSLG*	0.79	0.97	**1.17**	0.88	**1.08**	0.83
*KIF2C*	**−1.27**	−0.98	**−1.23**	−0.86	**−1.16**	−1.04
*MKI67*	**−1.00**	−0.68	−0.89	−0.71	−0.98	−0.72
*MXD1*	0.47	**1.00**	0.64	0.96	0.40	0.78
*PDGFA*	**1.00**	0.94	**1.12**	0.45	0.99	0.77
*POU3F2*	1.98	0.69	**2.89**	0.93	0.95	0.26
*RACGAP1*	**−1.18**	−0.93	**−1.11**	−0.86	**−1.06**	−0.82
*SERTAD1*	**1.08**	0.72	0.83	0.50	**1.01**	0.54
*TPX2*	**−1.01**	−0.87	−0.99	−0.87	−0.91	−0.78
*TTK*	**−1.05**	−0.74	−0.99	−0.74	−0.89	−0.65

^1^ N8, H8 and R8 signify irradiated normoxic, continuously hypoxic (1 % O_2_) and reoxygenated cells, respectively, while N0, H0 and R0 denote unirradiated normoxic cells, continuously hypoxic (1 % O_2_) and reoxygenated cells A549 cells. All cells were preincubated for 48 h in specified oxygen conditions, followed by irradiation and a medium change. RNA isolation from cells was carried out 4 h after irradiation. Statistically significant results are in bold; in red: upregulation, in blue: downregulation. The GSEA hallmark pathway for cell proliferation (M16210) was utilized to acquire proliferation-related reference genes.

**Table A9:** Effect of exposure to ionizing radiation on differential expression of EMT-related genes by normoxic A549 cells (N), continuously hypoxic (1 % O_2_) A549 cells (H), and hypoxic A549 cells reoxygenated after irradiation(R). Differential expression is evaluated against respective unirradiated controls.

NCBI Gene Symbol	Log_2_ Fold Change					
	N8 ^1^ vs. N0		H8 vs. H0		R8 vs. R0	
	X-Rays	Carbon Ions	X-Rays	Carbon Ions	X-Rays	Carbon Ions
*ACTA2*	0.98	**1.55**	**1.74**	**2.23**	0.99	**2.17**
*FAS*	**2.06**	**2.31**	**2.75**	**2.85**	**2.16**	**2.64**
*GADD45A*	**1.11**	0.77	0.95	0.55	**1.02**	0.91
*PDGFRB*	0.90	1.04	1.21	**1.96**	1.30	**1.81**

^1^ N8, H8, and R8 signify irradiated normoxic, continuously hypoxic (1 % O_2_), and reoxygenated cells, respectively, while N0, H0, and R0 denote unirradiated normoxic cells, continuously hypoxic (1 % O_2_), and reoxygenated A549 cells. All cells were preincubated for 48 h in specified oxygen conditions, followed by irradiation and a medium change. RNA isolation from cells was carried out 4 h after irradiation. Statistically significant results are in bold; in red: upregulation. The GSEA hallmark pathway for epithelial–mesenchymal transition (M5930) was utilized to acquire EMT reference genes.

## Appendix B

**Table A10:** List of all significant DEGs with complete names and primary functions.

NCBI Gene Symbol	Name	Primary Function (NCBI Gene Summary)
*ABI3BP*	ABI Family Member 3 Binding Protein	Enables actin filament binding activity. Predicted to be involved in extracellular matrix organization, positive regulation of cell–substrate adhesion, and regulation of post-synapse organization.
*ACTA2*	Actin Alpha 2	Encodes one of six actin proteins. Actins are involved in cell motility, structure, integrity, and intercellular signaling. The encoded protein is a smooth muscle actin that is involved in vascular contractility and blood pressure homeostasis.
*ADRA1D*	Adrenoceptor Alpha 1D	Alpha-1-adrenergic receptors (alpha-1-ARs) are members of the G protein-coupled receptor superfamily. They activate mitogenic responses and regulate growth and proliferation of many cells.
*ALDOC*	Aldolase, Fructose-Bisphosphate C	Encodes a member of the class I fructose-biphosphate aldolase gene family. Expressed specifically in the brain. It is a glycolytic enzyme that catalyzes the reversible cleavage of fructose-1,6-biphosphate and fructose 1-phosphate to dihydroxyacetone phosphate and either glyceraldehyde-3-phosphate or glyceraldehyde, respectively.
*ALOX12*	Arachidonate 12-Lipoxygenase, 12S Type	Encodes a member of the lipoxygenase protein family. It acts on different polyunsaturated fatty acid substrates to generate bioactive lipid mediators, including eicosanoids and lipoxins. It has been shown to regulate platelet function.
*BMPR2*	Bone Morphogenetic Protein Receptor Type 2	Encodes a member of the bone morphogenetic protein (BMP) receptor family of transmembrane serine/threonine kinases. The ligands of this receptor are members of the TGF-beta superfamily. BMPs are involved in endochondral bone formation and embryogenesis.
*BNC1*	Basonuclin Zinc Finger Protein 1	Encodes a zinc finger protein; thought to play a regulatory role in keratinocyte proliferation, and it may also be a regulator for rRNA transcription. Disruption of this gene has been implicated in premature ovarian failure as well as premature testicular aging.
*BPGM*	Bisphosphoglycerate Mutase	Encodes an enzyme that catalyzes 2,3-DPG synthesis via its synthetase activity and 2,3-DPG degradation via its phosphatase activity. Deficiency of this enzyme increases the affinity of cells for oxygen. Multiple alternatively spliced variants, encoding the same protein, have been identified.
*BTG2*	BTG Anti-Proliferation Factor 2	Encodes a member of the BTG/Tob family. This family has anti-proliferative properties. The encoded protein is involved in the regulation of the G1/S transition of the cell cycle.
*BUB1*	BUB1 Mitotic Checkpoint Serine/Threonine Kinase	Encodes a serine/threonine kinase that functions by activating the spindle checkpoint. This protein also plays a role in inhibiting the activation of the anaphase-promoting complex/cyclosome. It may also function in the DNA damage response. Mutations have been associated with aneuploidy and several forms of cancer.
*CCN2*	Cellular Communication Network Factor 2	Encodes a mitogen that is secreted by vascular endothelial cells. Plays a role in chondrocyte proliferation and differentiation and cell adhesion in many cell types and is related to platelet-derived growth factor.
*CD274*	CD274 Molecule	Encodes an immune inhibitory receptor ligand that is expressed by hematopoietic and non-hematopoietic cells, such as T cells and B cells and various types of tumor cells. Interaction of this ligand with its receptor inhibits T-cell activation and cytokine production. In tumor microenvironments, this interaction provides an immune escape for tumor cells through cytotoxic T-cell inactivation.
*CD70*	CD70 Molecule	Encodes a cytokine that belongs to the tumor necrosis factor (TNF) ligand family. It is a ligand for TNFRSF27/CD27. It induces proliferation of co-stimulated T cells, enhances the generation of cytolytic T cells, and contributes to T cell activation. This cytokine is also reported to play a role in regulating B-cell activation, the cytotoxic function of natural killer cells, and immunoglobulin synthesis.
*CDC25C*	Cell Division Cycle 25C	Encodes a protein that directs dephosphorylation of cyclin B-bound CDC2 and triggers entry into mitosis. It also suppresses p53-induced growth arrest.
*CDH6*	Cadherin 6	Encodes a type II cadherin. Cadherins are membrane glycoproteins that mediate homophilic cell–cell adhesion and play critical roles in cell differentiation and morphogenesis. It may play a role in kidney development as well as endometrium and placenta formation. Decreased expression may be associated with tumor growth and metastasis.
*CDKN1A*	Cyclin-Dependent Kinase Inhibitor 1A	Encodes a potent cyclin-dependent kinase inhibitor, which binds to and inhibits CDK2 and CDK4 complexes, and thus functions as a regulator of cell cycle progression at G1. The expression of this gene is tightly controlled by the tumor suppressor protein p53, through which this protein mediates the p53-dependent cell cycle G1 phase arrest in response to a variety of stress stimuli. This protein can interact with PCNA, a DNA polymerase accessory factor, and plays a regulatory role in DNA replication and DNA damage repair. CDKN1A was reported to be cleaved by CASP3-like caspases, which leads to activation of CDK2 and may be instrumental in the execution of apoptosis.
*CDKN2C*	Cyclin-Dependent Kinase Inhibitor 2C	Encodes a member of the INK4 family of cyclin-dependent kinase inhibitors. It binds to and inhibits CDK4 or CDK6, thus regulating cell growth by controlling cell cycle G1 progression. Ectopic expression of this gene was shown to suppress the growth of human cells in a manner that appears to correlate with the presence of a wild-type RB1 function. It may have a role in regulating spermatogenesis, as well as in suppressing tumorigenesis.
*CHRM1*	Cholinergic Receptor Muscarinic 1	Involved in the mediation of bronchoconstriction and in the acid secretion of the gastrointestinal tract. The functional diversity of such receptors includes cellular responses such as adenylate cyclase inhibition, phosphoinositide degeneration, and potassium channel mediation.
*COL1A1*	Collagen Type I Alpha 1 Chain	Encodes the pro-alpha1 chains of type I collagen found in most connective tissues. Reciprocal translocations between chromosomes 17 and 22, where this gene and the gene for PDGF-beta are located, are associated with a skin tumor called dermatofibrosarcoma protuberans, resulting from unregulated expression of the growth factor.
*COL4A1*	Collagen Type IV Alpha 1 Chain	Encodes a type IV collagen alpha protein, an integral component of basement membranes. The protein interacts with other extracellular matrix components such as perlecans, proteoglycans, and laminins. In addition, proteolytic cleavage of the non-collagenous carboxy-terminal domain results in a biologically active fragment known as arresten, which has anti-angiogenic and tumor suppressor properties.
*COL5A1*	Collagen Type V Alpha 1 Chain	Encodes an alpha chain for one of the low-abundance fibrillar collagens. Type V collagen is found in tissues containing type I collagen and appears to regulate the assembly of heterotypic fibers composed of both type I and type V collagen. This gene product is closely related to type XI collagen.
*COL6A3*	Collagen Type VI Alpha 3 Chain	Encodes the alpha-3 chain of type VI collagen, a beaded filament collagen found in most connective tissues. Its domains have been shown to bind extracellular matrix proteins, an interaction that explains the importance of this collagen in organizing matrix components.
*CSF1*	Colony-Stimulating Factor 1	Encodes a cytokine that controls the production, differentiation, and function of macrophages.
*CTF1*	Cardiotrophin 1	Encodes a secreted cytokine that induces cardiac myocyte hypertrophy in vitro.
*CTHRC1*	Collagen Triple Helix Repeat-Containing 1	Encodes a protein that may play a role in the cellular response to arterial injury through involvement in vascular remodeling.
*CXCL2*	C-X-C Motif Chemokine Ligand 2	Encodes a secreted CXC-chemokine involved in immunoregulatory and inflammatory processes. It may suppress hematopoietic progenitor cell proliferation.
*CXCL8*	C-X-C Motif Chemokine Ligand 8	Encodes a CXC-chemokine family and is a major mediator of the inflammatory response. It is secreted by mononuclear macrophages, neutrophils, eosinophils, T lymphocytes, epithelial cells, and fibroblasts. It functions as a chemotactic factor for neutrophils. Plays a role in the pathogenesis of the lower respiratory tract infection bronchiolitis, a common respiratory tract disease. The overproduction of this pro-inflammatory protein is thought to cause the lung inflammation associated with cystic fibrosis. This protein is also secreted by tumor cells and promotes tumor migration, invasion, angiogenesis, and metastasis. It is also a potent angiogenic factor.
*DLEC1*	DLEC1 Cilia and Flagella-Associated Protein	It is located in a region that is commonly deleted in a variety of malignancies. Down-regulation of this gene has been observed in several human cancers, including lung cancer.
*EBI3*	Epstein–Barr Virus-Induced 3	Encodes a secreted glycoprotein belonging to the hematopoietin receptor family and heterodimerizes with a 28 kDa protein to form interleukin 27 (IL-27). IL-27 regulates T cell and inflammatory responses.
*EHF*	ETS Homologous Factor	Encodes a protein that belongs to an ETS transcription factor subfamily characterized by epithelial-specific expression (ESEs). The encoded protein acts as a transcriptional repressor and may be involved in epithelial differentiation and carcinogenesis.
*ENO2*	Enolase 2	Encodes one of the three enolase isoenzymes involved in glycolysis found in mammals. This isoenzyme, a homodimer, is found in mature neurons and cells of neuronal origin.
*ETS1*	ETS Proto-Oncogene 1, Transcription Factor	This gene encodes a member of the ETS family of transcription factors and functions either as transcriptional activators or repressors of numerous genes and is involved in stem cell development, cell senescence and death, and tumorigenesis.
*FABP6*	Fatty Acid Binding Protein 6	Encodes the ileal fatty acid binding protein that binds long-chain fatty acids and other hydrophobic ligands. FABP’s roles include fatty acid uptake, transport, and metabolism.
*FAS*	Fas Cell Surface Death Receptor	The protein encoded by this gene is a member of the TNF-receptor superfamily. This receptor contains a death domain. It has been shown to play a central role in the physiological regulation of programmed cell death and has been implicated in the pathogenesis of various malignancies and diseases of the immune system. This receptor has also been shown to activate NF-κB, MAPK3/ERK1, and MAPK8/JNK and is found to be involved in transducing the proliferating signals in normal diploid fibroblast and T cells.
*FBLN2*	Fibulin 2	Encodes an extracellular matrix protein, which belongs to the fibulin family. This protein binds various extracellular ligands and calcium. It may play a role during organ development, in particular, during the differentiation of heart, skeletal, and neuronal structures.
*FGF18*	Fibroblast Growth Factor 18	Encodes a member of the FGF family, which possesses broad mitogenic and cell survival activities and is involved in a variety of biological processes, including embryonic development, cell growth, morphogenesis, tissue repair, tumor growth, and invasion. This protein is a pleiotropic growth factor that stimulates proliferation in a number of tissues, most notably the liver and small intestine.
*FIGF (VEGFD)*	Vascular Endothelial Growth Factor D	Encodes a member of the PDGF/VEGF family and is active in angiogenesis, lymphangiogenesis, and endothelial cell growth. It binds and activates VEGFR-2 and VEGFR-3. It is structurally and functionally similar to VEGFC.
*FSTL1*	Follistatin Like 1	Encodes a protein with similarity to follistatin, an activin-binding protein. It is an autoantigen associated with rheumatoid arthritis.
*GADD45A and GADD45B*	Growth Arrest and DNA Damage Inducible Alpha and Beta	They are members of a group of genes that are upregulated following stressful growth arrest conditions and treatment with DNA-damaging agents. Mediate activation of the p38/JNK pathway via MTK1/MEKK4 kinase. Their upregulation is mediated by both p53-dependent and -independent mechanisms.
*HK2*	Hexokinase2	Hexokinases phosphorylate glucose to produce glucose-6-phosphate, the first step in most glucose metabolism pathways. HK2 is the predominant form found in skeletal muscle.
*ICOSLG*	Inducible T Cell Costimulator Ligand	Enables identical protein binding activity. Predicted to be involved in the T cell receptor signaling pathway and positive regulation of interleukin-4 production. Located in intracellular membrane-bounded organelles and plasma membrane.
*IGFBP3*	Insulin Like Growth Factor Binding Protein 3	A member of the insulin-like growth factor binding protein (IGFBP) family. It circulates in the plasma, prolonging the half-life of IGFs and altering their interaction with cell surface receptors.
*IGFBP7*	Insulin Like Growth Factor Binding Protein 7	Encodes a member of the IGFBP family and regulates IGF availability in body fluids and tissues and modulates IGF binding to its receptors. This protein binds IGF-I and IGF-II with relatively low affinity and belongs to a subfamily of low-affinity IGFBPs. It also stimulates prostacyclin production and cell adhesion.
*IL1A*	Interleukin 1 Alpha	Encodes a member of the interleukin 1 cytokine family. It is a pleiotropic cytokine involved in various immune responses, inflammatory processes, and hematopoiesis. This cytokine is produced by monocytes and macrophages as a proprotein, which is proteolytically processed and released in response to cell injury, and thus induces apoptosis.
*IL1B*	Interleukin 1 Beta	Encodes a member of the interleukin 1 cytokine family. This cytokine is produced by activated macrophages as a proprotein, which is proteolytically processed to its active form by caspase 1 (CASP1/ICE). This cytokine is an important mediator of the inflammatory response and is involved in a variety of cellular activities, including cell proliferation, differentiation, and apoptosis. Patients with severe Coronavirus Disease 2019 (COVID-19) present elevated levels of pro-inflammatory cytokines such as IL-1B in bronchial alveolar lavage fluid samples.
*IL31RA*	Interleukin 31 Receptor A	Encodes a protein of the type I cytokine receptor family expressed on monocytes and is involved in IL-31 signaling via activation of STAT-3 and STAT-5. It functions either as a monomer or as part of a receptor complex with oncostatin M receptor (OSMR).
*IL32*	Interleukin 32	Expression of this protein is increased after the activation of T-cells by mitogens or the activation of NK cells by IL-2. This protein induces the production of TNFα from macrophage cells.
*IL6*	Interleukin 6	Encodes a cytokine that functions in inflammation and the maturation of B cells. It is an endogenous pyrogen. It is primarily produced at sites of acute and chronic inflammation, where it is secreted into the serum and induces a transcriptional inflammatory response through interleukin 6 receptor, alpha. Elevated levels of the encoded protein have been found in virus infections, including COVID-19.
*IL6R*	Interleukin 6 Receptor	Encodes a subunit of the interleukin 6 (IL6) receptor complex. Dysregulated production of IL6 and this receptor are implicated in the pathogenesis of many diseases, such as multiple myeloma, autoimmune diseases, and prostate cancer.
*INHBA*	Inhibin Subunit Beta A	Encodes a member of the TGF-beta superfamily of proteins. Inhibits follicle-stimulating hormone secretion. It also plays a role in eye, tooth, and testis development. Elevated expression of this gene may be associated with cancer cachexia in human patients.
*ITGA2*	Integrin Subunit Alpha 2	Encodes the alpha subunit of a transmembrane receptor for collagens and related proteins. The encoded protein forms a heterodimer with a beta subunit and mediates the adhesion of platelets and other cell types to the extracellular matrix.
*KIF2C*	Kinesin Family Member 2C	Encodes a kinesin-like protein that functions as a microtubule-dependent molecular motor. The encoded protein can depolymerize microtubules at the plus end, thereby promoting mitotic chromosome segregation.
*KITLG*	KIT Ligand	Encodes the ligand of the tyrosine-kinase receptor encoded by the KIT locus. This ligand is a pleiotropic factor that acts in utero in germ cell and neural cell development and hematopoiesis, all believed to reflect a role in cell migration.
*LAMC2*	Laminin Subunit Gamma 2	This gene encodes the gamma chain isoform laminin, gamma 2. It is expressed in several fetal tissues but differently from gamma 1 and is specifically localized to epithelial cells in skin, lung, and kidney. The gamma 2 chain together with the alpha 3 and beta 3 chains constitute laminin 5 (earlier known as kalinin), which is an integral part of the anchoring filaments that connect epithelial cells to the underlying basement membrane. Laminins are the major non-collagenous constituent of basement membranes. They are implicated in a wide variety of biological processes, including cell adhesion, differentiation, migration, signaling, neurite outgrowth, and metastasis.
*LDHA*	Lactate Dehydrogenase A	Encodes the A subunit of LDH, which catalyzes the reversible conversion of pyruvate to lactate with the concomitant oxidation of NADH to NAD in anaerobic glycolysis.
*LMO1*	LIM Domain Only 1	This locus encodes a transcriptional regulator that contains two cysteine-rich LIM domains but lacks a DNA-binding domain. LIM domains may play a role in protein interactions; thus, the encoded protein may regulate transcription by competitively binding to specific DNA-binding transcription factors.
*LOX*	Lysyl Oxidase	This gene encodes a member of the lysyl oxidase family of proteins. The copper-dependent amine oxidase activity of this enzyme functions in the crosslinking of collagens and elastin.
*LOXL1*	Lysyl Oxidase Like 1	This gene encodes a member of the lysyl oxidase family of proteins. The prototypic member of the family is essential to the biogenesis of connective tissue, encoding an extracellular copper-dependent amine oxidase that catalyzes the first step in the formation of crosslinks in collagen and elastin. The encoded preproprotein is proteolytically processed to generate the mature enzyme.
*LOXL2*	Lysyl Oxidase Like 2	This gene encodes a member of the lysyl oxidase gene family. The prototypic member of the family is essential to the biogenesis of connective tissue, encoding an extracellular copper-dependent amine oxidase that catalyzes the first step in the formation of crosslinks in collagens and elastin.
*LRRC15*	Leucine-Rich Repeat Containing 15	Enables several functions, including fibronectin binding activity, laminin binding activity, and protein sequestering activity. Involved in several processes, including negative regulation of protein localization to the plasma membrane; negative regulation of viral entry into the host cell; and receptor-mediated virion attachment to host cell. Located in the collagen-containing extracellular matrix and the plasma membrane. It is active in the apical plasma membrane.
*MATN3*	Matrilin 3	This gene encodes a member of the Von Willebrand factor A domain-containing protein family. This family of proteins is thought to be involved in the formation of filamentous networks in the extracellular matrices of various tissues. This protein contains two von Willebrand factor A domains; it is present in the cartilage extracellular matrix and has a role in the development and homeostasis of cartilage and bone. Mutations in this gene result in multiple epiphyseal dysplasia.
*MDM2*	MDM2 Proto-Oncogene	This gene encodes a nuclear-localized E3 ubiquitin ligase. The encoded protein can promote tumor formation by targeting tumor suppressor proteins, such as p53, for proteasomal degradation. This gene is itself transcriptionally regulated by p53. Overexpression or amplification of this locus is detected in a variety of different cancers. There is a pseudogene for this gene on chromosome 2. Alternative splicing results in a multitude of transcript variants, many of which may be expressed only in tumor cells.
*MKI67*	Marker of Proliferation Ki-67	Enables RNA binding activity. Involved in regulation of chromosome segregation and regulation of mitotic nuclear division. Located in condensed chromosomes, nuclear bodies, and nucleoli. Implicated in several diseases, including Crohn’s disease, colorectal cancer, endocrine gland cancer (multiple), graft-versus-host disease, and human immunodeficiency virus infectious disease. Biomarker of several diseases, including Barrett’s esophagus; autoimmune disease of the musculoskeletal system (multiple); endocrine gland cancer (multiple); gastrointestinal system cancer (multiple); and lung cancer (multiple).
*MXD1*	MAX Dimerization Protein 1	This gene encodes a member of the MYC/MAX/MAD network of basic helix-loop-helix leucine zipper transcription factors. The MYC/MAX/MAD transcription factors mediate cellular proliferation, differentiation, and apoptosis. The encoded protein antagonizes MYC-mediated transcriptional activation of target genes by competing for the binding partner MAX and recruiting repressor complexes containing histone deacetylases. Mutations in this gene may play a role in acute leukemia, and the encoded protein is a potential tumor suppressor. Alternatively spliced transcript variants encoding multiple isoforms have been observed for this gene.
*MYL9*	Myosin Light Chain 9	Myosin, a structural component of muscle, consists of two heavy chains and four light chains. The protein encoded by this gene is a myosin light chain that may regulate muscle contraction by modulating the ATPase activity of myosin heads. The encoded protein binds calcium and is activated by myosin light chain kinase. Two transcript variants encoding different isoforms have been found for this gene.
*PDGFA*	Platelet-Derived Growth Factor Subunit A	This gene encodes a member of the protein family comprising both platelet-derived growth factors (PDGF) and vascular endothelial growth factors (VEGF). The encoded preproprotein is proteolytically processed to generate platelet-derived growth factor subunit A, which can homodimerize or, alternatively, heterodimerize with the related platelet-derived growth factor subunit B. These proteins bind and activate PDGF receptor tyrosine kinases, which play a role in a wide range of developmental processes. Alternative splicing results in multiple transcript variants.
*PDGFRB*	Platelet-Derived Growth Factor Receptor Beta	The protein encoded by this gene is a cell surface tyrosine kinase receptor for members of the platelet-derived growth factor family. These growth factors are mitogens for cells of mesenchymal origin. The identity of the growth factor bound to a receptor monomer determines whether the functional receptor is a homodimer (PDGFB or PDGFD) or a heterodimer (PDGFA and PDGFB). This gene is essential for normal development of the cardiovascular system and aids in rearrangement of the actin cytoskeleton. This gene is flanked on chromosome 5 by the genes for granulocyte-macrophage colony-stimulating factor and macrophage-colony stimulating factor receptor; all three genes may be implicated in the 5-q syndrome. A translocation between chromosomes 5 and 12 that fuses this gene to that of the ETV6 gene results in chronic myeloproliferative disorder with eosinophilia.
*PGF*	Placental Growth Factor	Enables growth factor activity. Involved in positive regulation of cell population proliferation. Predicted to be located in the extracellular region. Predicted to be active in the extracellular space. Implicated in several diseases, including brain ischemia, diabetic neuropathy, glioblastoma, myocardial infarction, and pancreatic endocrine carcinoma. Biomarker of several diseases, including artery disease (multiple), autoimmune disease of musculoskeletal system (multiple), epilepsy (multiple), limited scleroderma, and pancreatic endocrine carcinoma.
*PGK1*	Phosphoglycerate Kinase 1	The protein encoded by this gene is a glycolytic enzyme that catalyzes the conversion of 1,3-diphosphoglycerate to 3-phosphoglycerate. The encoded protein may also act as a cofactor for polymerase alpha. Additionally, this protein is secreted by tumor cells, where it participates in angiogenesis by functioning to reduce disulfide bonds in the serine protease, plasmin, which consequently leads to the release of the tumor blood vessel inhibitor angiostatin. The encoded protein has been identified as a moonlighting protein based on its ability to perform mechanistically distinct functions. Deficiency of the enzyme is associated with a wide range of clinical phenotypes, including hemolytic anemia and neurological impairment. Pseudogenes of this gene have been defined on chromosomes 19, 21, and the X chromosome.
*PLK1*	Polo Like Kinase 1	The Ser/Thr protein kinase encoded by this gene belongs to the CDC5/Polo subfamily. It is highly expressed during mitosis, and elevated levels are found in many different types of cancer. Depletion of this protein in cancer cells dramatically inhibited cell proliferation and induced apoptosis; hence, it is a target for cancer therapy.
*PMEPA1*	Prostate Transmembrane Protein, Androgen Induced 1	This gene encodes a transmembrane protein that contains a Smad interacting motif (SIM). Expression of this gene is induced by androgens and transforming growth factor beta, and the encoded protein suppresses the androgen receptor and transforming growth factor beta signaling pathways through interactions with Smad proteins. Overexpression of this gene may play a role in multiple types of cancer. Alternatively spliced transcript variants encoding multiple isoforms have been observed for this gene.
*POU3F2*	POU Class 3 Homeobox 2	This gene encodes a member of the POU-III class of neural transcription factors. The encoded protein is involved in neuronal differentiation and enhances the activation of corticotropin-releasing hormone-regulated genes. Overexpression of this protein is associated with an increase in the proliferation of melanoma cells.
*PPM1D*	Protein Phosphatase, Mg^2+^/Mn^2+^ Dependent 1D	The protein encoded by this gene is a member of the PP2C family of Ser/Thr protein phosphatases. PP2C family members are known to be negative regulators of cell stress response pathways. The expression of this gene is induced in a p53-dependent manner in response to various environmental stresses. While being induced by tumor suppressor protein TP53/p53, this phosphatase negatively regulates the activity of p38 MAP kinase, MAPK/p38, through which it reduces the phosphorylation of p53, and in turn suppresses p53-mediated transcription and apoptosis. This phosphatase thus mediates a feedback regulation of p38-p53 signaling that contributes to growth inhibition and the suppression of stress-induced apoptosis. This gene is located in a chromosomal region known to be amplified in breast cancer. The amplification of this gene has been detected in both breast cancer cell lines and primary breast tumors, which suggests a role of this gene in cancer development.
*PTK2B*	Protein Tyrosine Kinase 2 Beta	This gene encodes a cytoplasmic protein tyrosine kinase, which is involved in calcium-induced regulation of ion channels and activation of the map kinase signaling pathway. The encoded protein may represent an important signaling intermediate between neuropeptide-activated receptors or neurotransmitters that increase calcium flux and the downstream signals that regulate neuronal activity. The encoded protein undergoes rapid tyrosine phosphorylation and activation in response to increases in the intracellular calcium concentration, nicotinic acetylcholine receptor activation, membrane depolarization, or protein kinase C activation. This protein has been shown to bind CRK-associated substrate, nephrocystin, GTPase regulator associated with FAK, and the SH2 domain of GRB2. The encoded protein is a member of the FAK subfamily of protein tyrosine kinases but lacks significant sequence similarity to kinases from other subfamilies. Four transcript variants encoding two different isoforms have been found for this gene.
*RACGAP1*	Rac GTPase Activating Protein 1	This gene encodes a GTPase-activating protein (GAP) that is a component of the central spindlin complex. This protein binds activated forms of Rho GTPases and stimulates GTP hydrolysis, which results in negative regulation of Rho-mediated signals. This protein plays a regulatory role in cytokinesis, cell growth, and differentiation. Alternatively spliced transcript variants have been found for this gene. There is a pseudogene for this gene on chromosome 12.
*SCG2*	Secretogranin II	The protein encoded by this gene is a member of the chromogranin/secretogranin family of neuroendocrine secretory proteins. Studies in rodents suggest that the full-length protein, secretogranin II, is involved in the packaging or sorting of peptide hormones and neuropeptides into secretory vesicles. The full-length protein is cleaved to produce the active peptide secretoneurin, which exerts chemotaxic effects on specific cell types, and EM66, whose function is unknown.
*SERPINE1*	Serpin Family E Member 1	Encodes a member of the serpin superfamily. This member is the principal inhibitor of tissue plasminogen activator (tPA) and urokinase (uPA) and hence is an inhibitor of fibrinolysis.
*SERTAD1*	SERTA Domain Containing 1	Acts upstream of or within negative regulation of cell growth. Located in cytoplasm and nucleus.
*SESN1*	Sestrin 1	This gene encodes a member of the sestrin family. Sestrins are induced by the p53 tumor suppressor protein and play a role in the cellular response to DNA damage and oxidative stress. The encoded protein mediates p53 inhibition of cell growth by activating AMP-activated protein kinase, which results in the inhibition of the mammalian target of rapamycin protein. The encoded protein also plays a critical role in antioxidant defense by regenerating overoxidized peroxiredoxins, and the expression of this gene is a potential marker for exposure to radiation. Alternatively spliced transcript variants encoding multiple isoforms have been observed for this gene.
*SNAI2*	Snail Family Transcriptional Repressor 2	This gene encodes a member of the Snail family of C2H2-type zinc finger transcription factors. The encoded protein acts as a transcriptional repressor that binds to E-box motifs and is also likely to repress E-cadherin transcription in breast carcinoma. This protein is involved in epithelial–mesenchymal transitions and has antiapoptotic activity. Mutations in this gene may be associated with sporadic cases of neural tube defects.
*SPOCK1*	SPARC (Osteonectin), Cwcv, and Kazal-Like Domains Proteoglycan 1	This gene encodes the protein core of a seminal plasma proteoglycan containing chondroitin- and heparan-sulfate chains. The protein’s function is unknown, although similarity to thyropin-type cysteine protease inhibitors suggests its function may be related to protease inhibition.
*SSTR5*	Somatostatin Receptor 5	The protein encoded by this gene is one of the SSTRs, which is a multi-pass membrane protein. The activity of this receptor is mediated by G proteins, which inhibit adenylyl cyclase, and different regions of this receptor molecule are required for the activation of different signaling pathways.
*SYK*	Spleen Associated Tyrosine Kinase	Encodes a member of the family of non-receptor-type Tyr protein kinases. This protein is widely expressed in hematopoietic cells and is involved in coupling activated immunoreceptors to downstream signaling events that mediate diverse cellular responses, including proliferation, differentiation, and phagocytosis. It is thought to be a modulator of epithelial cell growth and a potential tumor suppressor in human breast carcinomas.
*TAGLN*	Transgelin	Encodes a shape-change- and transformation-sensitive actin-binding protein that belongs to the calponin family. It is an early marker of smooth muscle differentiation. Involved in calcium-independent smooth muscle contraction. It acts as a tumor suppressor, and the loss of its expression is an early event in cell transformation and the development of some tumors.
*TGFBI*	Transforming Growth Factor Beta Induced	Encodes an RGD-containing protein that binds to type I, II, and IV collagens. The RGD motif is found in many ECM proteins modulating cell adhesion and serves as a ligand recognition sequence for several integrins. Plays a role in cell-collagen interactions. Induced by transforming growth factor-beta and acts to inhibit cell adhesion.
*TGFBR3*	Transforming Growth Factor Beta Receptor 3	The encoded receptor is a membrane proteoglycan that often functions as a co-receptor with other TGF-beta receptor superfamily members. Ectodomain shedding produces soluble TGFBR3, which may inhibit TGFB signaling. Decreased expression of this receptor has been observed in various cancers.
*TIMP3*	TIMP Metallopeptidase Inhibitor 3	The family encodes inhibitors of the MMPs. Induced in response to mitogenic stimulation.
*TNFSF9*	TNF Superfamily Member 9	Encodes a cytokine that belongs to the TNF ligand family that acts as a ligand for TNFRSF9/4-1BB, which is a costimulatory receptor molecule in T lymphocytes. This cytokine and its receptor are involved in the antigen presentation process and in the generation of cytotoxic T cells. It has been shown to reactivate anergic T lymphocytes in addition to promoting T lymphocyte proliferation. It is expressed in carcinoma cell lines and is thought to be involved in T cell–tumor cell interaction.
*TPM1*	Tropomyosin 1	A member of the tropomyosin family of actin-binding proteins involved in the contractile system of striated and smooth muscles. In non-muscle cells, it is implicated in stabilizing cytoskeleton actin filaments.
*TPX2*	TPX2 Microtubule Nucleation Factor	Involved in activation of protein kinase activity, microtubule cytoskeleton organization, and negative regulation of microtubule depolymerization. Located in the intercellular bridge, nucleoplasm, and spindle.
*TTK*	TTK Protein Kinase	Encodes a dual specificity protein kinase associated with cell proliferation; this protein is essential for chromosome alignment at the centromere during mitosis and is required for centrosome duplication. It has been found to be a critical mitotic checkpoint protein for accurate segregation of chromosomes during mitosis. Tumorigenesis may occur when this protein fails to degrade and produces excess centrosomes, resulting in aberrant mitotic spindles.
*VEGFA*	Vascular Endothelial Growth Factor A	Encodes a member of the PDGF/VEGF growth factor family. This growth factor induces proliferation and migration of vascular endothelial cells and is essential for both physiological and pathological angiogenesis. This gene is upregulated in many known tumors, and its expression is correlated with tumor stage and progression. The levels of VEGF are increased during infection with SARS-CoV-2, thus promoting inflammation by facilitating recruitment of inflammatory cells and by increasing the level of angiopoietin II (Ang II). In turn, Ang II facilitates the elevation of VEGF, thus forming a vicious cycle in the release of inflammatory cytokines.
*WNT5A*	WNT Family Member 5 A	Encodes a secreted signaling protein that has been implicated in embryogenesis as well as in oncogenesis. It decreases proliferation, migration, invasiveness, and clonogenicity of carcinoma cells and may act as a tumor suppressor.
